# Organic Chemistry as a Catalyst for AI Innovation: Challenges, Methods, and Emerging Paradigms

**DOI:** 10.1021/acs.chemrev.5c01081

**Published:** 2026-06-17

**Authors:** Nitesh V. Chawla, Gisela A. González-Montiel, Kehan Guo, Taicheng Guo, Ting Hua, Xiaobao Huang, Eric Inae, Meng Jiang, Khiem Le, Gang Liu, J. Charlie Maier, Nuno Moniz, Brenda Nogueira, Deng Pan, Bryan V. Piguave, Brett M. Savoie, Andrew B. Schofield, Yili Shen, Alexander Taylor, Xiangliang Zhang, Yihan Zhu, Olaf Wiest

**Affiliations:** † Department of Computer Science and Engineering, 6111 University of Notre Dame, Fitzpatrick Hall of Engineering, Notre Dame, Indiana 46556, United States; ‡ Lucy Family Institute for Data and Society, 6111 University of Notre Dame, 384 Nieuwland Science Hall, Notre Dame, Indiana 46556, United States; ¶ Department of Chemistry and Biochemistry, 6111 University of Notre Dame, 236 Nieuwland Science Hall, Notre Dame, Indiana 46556, United States; § Department of Chemical and Biomolecular Engineering, 6111 University of Notre Dame, 250 Nieuwland Science Hall, Notre Dame, Indiana 46556, United States

## Abstract

Artificial intelligence and organic chemistry are redefining each other in a fundamentally bidirectional relationship. This Review highlights how the intrinsic challenges of organic chemistry have acted as a catalyst for conceptual and methodological innovation in AI itself. Sparse and heterogeneous reaction data sets spurred the development of self-supervised and few-shot learning paradigms; the combinatorial complexity of multireactant chemistry motivated the transition from graph neural networks to hypergraph architectures; the need to bridge symbolic chemical reasoning with statistical prediction inspired chemical language models grounded in large language model frameworks; and the iterative, decision-intensive nature of synthesis planning catalyzed the rise of autonomous agentic systems. We survey the multimodal landscape of chemical data, tracing the evolution of molecular representations from classical fingerprints to geometric encodings and examining how each representation class shapes downstream model capabilities. We analyze how data scarcity and uneven property distributions have driven advances in transfer learning, self-supervised pretraining, and meta-learning frameworks tailored to molecules and reactions. Reaction prediction, mechanistic inference, and retrosynthesis planning are examined as core areas where chemistry has shaped modern AI techniques. We further explore chemical reasoning through multimodal fusion, generative molecular design, and self-driving laboratories. We conclude by identifying persistent challenges, including data sparsity, selection bias, benchmark-to-lab gaps, and reproducibility.

## Graphical abstract

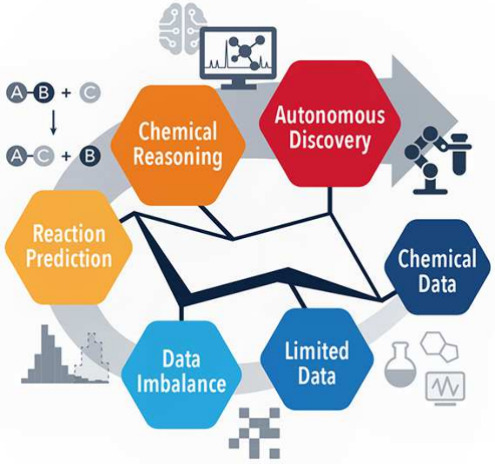

## 1 Introduction

The convergence of artificial intelligence (AI) and organic chemistry represents one of the most transformative developments in modern science. While much of the dialogue has centered on how AI is advancing scientific discovery, this Review presents a reverse narrative: it examines how the intrinsic challenges of organic chemistry have served as powerful catalysts for new conceptual and methodological innovation in AI. Consider the development trajectories of a few recent methods: sparse reaction databases necessitated few-shot and self-supervised learning paradigms; multireactant complexity drove the evolution from graph neural networks to hypergraph neural networks; the need to bridge symbolic chemistry and predictive modeling led to chemical language models predicated on the modern day advances in large language models (LLMs); and the iterative, decision-driven nature of synthesis planning inspired autonomous agentic frameworks.

The decision to focus this Review on the influence of chemistry in the evolution of AI means that we will not review the large number of important counterdiffusions from AI into chemistry. This decision does not diminish the importance of bidirectional influence; rather, it is a deliberate trade-off that we are making in recognition of the many excellent recent reviews of AI in chemistry. This reframing positions scientific domains as engines of algorithmic progress in AI and underscores the value of cross-disciplinary feedback loops. Understanding how chemical problems induce generalizable AI solutions not only highlights past advances but also points toward future opportunities for synergy between physical sciences and AI innovation. At the same time, it introduces chemists to recent developments in AI that serve to advance data chemistry but are often published in computer science conferences and preprints rather than the chemical literature. This Perspective is essential as AI systems expand their role in scientific discovery, and as scientific fields continue to present novel, structurally rich, and intellectually demanding problems that challenge the boundaries of existing AI methodologies.

### 1.1 Scope and Organization

This Review is structured to provide a comprehensive examination of how the challenges inherent to organic chemistry have motivated advances in AI up to October 2025. The organization of the paper follows the conceptual progression from foundational data issues to increasingly sophisticated algorithmic and agentic systems, mirroring both the evolution of AI methods and the logical dependencies between them.

- Section 2 addresses the fundamental data challenges that arise in organic chemistry, including heterogeneity across modalities, diverse molecular representations, and the integration of symbolic, structural, and spectroscopic information. We examine how chemistry’s multimodal data landscape has driven the development of cross-modal pretraining architectures and domain-adaptive fusion strategies that extend beyond chemistry into broader multimodal AI research.
- Section 3 focuses on learning with limited data, examining how chemistry’s extreme data scarcity has necessitated algorithmic innovations in transfer learning, self-supervised pretraining, meta-learning, and foundation models. We trace how these paradigms for extracting maximum value from small labeled data sets have emerged from chemistry’s challenges and now inform low-resource learning across scientific domains.
- Section 4 explores data rebalancing and generation strategies that address both imbalanced regression and synthetic data augmentation. We examine how chemistry’s long-tailed property distributions and the need to generate chemically valid molecules under sparse supervision have driven methodological advances in relevance-weighted losses, structure-space generation, and physics-informed augmentation that balance fidelity to chemical constraints with exploration of underrepresented regions.
- Section 5 covers reaction prediction and retrosynthesis, topics that have historically served as early testbeds for AI in chemistry. We trace the progression from rule-based expert systems to modern data-driven models, highlighting how the combinatorial complexity of multireactant interactions and exponential retrosynthetic search spaces have spurred the development of hypergraph neural networks, reaction-center attention mechanisms, Monte Carlo tree search with learned guidance, and reinforcement learning frameworks for multistep planning.
- Section 6 explores chemical reasoning, emphasizing the shift from inductive pattern recognition to deductive mechanistic inference. This section reviews how chemistry’s ill-posed inverse problems and one-to-many mappings have driven the development of structural generators (from variational autoencoders (VAEs) to diffusion models), multimodal fusion architectures, constraint-satisfaction layers, and physics-informed neural networks that reason backward from effects to causes under incomplete information.
- Section 7 addresses the emergence of agentic AI for autonomous chemical discovery. We define the core components of chemical AI agents, describe the architecture of self-driving laboratories, and analyze how the iterative, decision-intensive nature of chemical experimentation has driven frameworks integrating planning, execution, adaptive learning, and safety-critical operation in closed-loop workflows with physical laboratory instrumentation.
- Section 8 concludes this Review with a perspective on the progress to date, outstanding challenges, and opportunities for future research at the intersection of AI and organic chemistry. We discuss pathways toward unified chemical intelligence, emphasizing the need for synergistic development of data infrastructure, model architectures, multimodal reasoning, and autonomous experimentation.

Together, these sections provide a structured view of how the scientific, representational, and operational challenges of organic chemistry have informed and accelerated the development of new AI frameworks, and how these advances, in turn, open new directions for chemical discovery. As AI systems expand into physics, biology, and materials science, the lessons learned from chemistry’s constraints will prove increasingly essential for building robust, interpretable, and scientifically grounded intelligent systems.

## 2 The Landscape of Chemical Data

Computational chemistry now spans text-based lab notebooks, pictures of spectra, microscopy videos, and molecular graphs (Figure 1). Each modality arrives with a different sampling density, metadata standard, and noise pattern, so assembling a unified training set rarely yields the volume or consistency expected by modern AI models.^1,2^ Deep networks are especially sensitive to these gaps: they require large, annotated corpora,^3–5^ yet most laboratories generate data sporadically and with instrument-specific quirks. Even high-throughput experimentation (HTE) pipelines mitigate only part of the variance—dosing errors, stirring efficiency, and sensor drift still leak into the records and must be probed via perturbation studies before trusting downstream models.^6–10^

**Figure 1:**
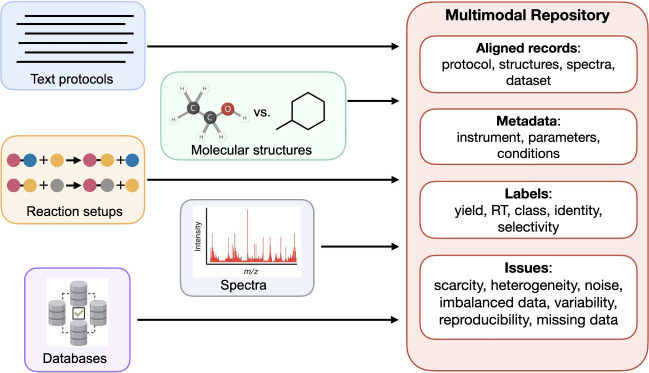
Multifaceted nature of chemical data in organic chemistry.

Chemistry’s use of AI has steadily moved from the rule-based expert systems of the 1960s to today’s data-driven pipelines,^11,12^ but the underlying data sets remain sparse, slow, and expensive to curate.^13^ Routine preprocessing (imputation, scaling, PCA) suppresses some heterogeneity,^14^ yet large fractions of spectra and chromatograms are still “dark matter” with no structural assignment.^15^ Yield measurements provide another reminder: most published reactions cluster in the 0–70% window, producing a lopsided evidence base that complicates reproducibility despite best-practice guidelines from cheminformatics groups.^13^

Meaningful molecular descriptors remain the bridge between chemistry and machine learning (ML). When retention times or other physicochemical properties are paired with well-defined descriptors, regression models can interpolate reliably to new structures.^16^ Progress on this front has come as much from improved experimental compilations and conformer generation to capture molecular flexibility as from algorithmic tweaks.

Recent deep-learning work has demonstrated tangible gains in spectral analysis: Convolutional Neural Networks (CNNs) separate true peaks from noise by exploiting peak shape and signal-to-noise ratios, while other architectures capture cross-modal context from paired spectra and structures.^11,17–20^ These models still require rigorous validation against experimentally confirmed (and frequently manually assigned) structures before they are trusted in production workflows.^15^ As reference libraries and compute budgets grow, similar strategies are spilling into domains such as small-molecule high-throughput screening, but the same data sparsity concerns persist.^21^

### 2.1 Molecular Representations for Machine Learning

Given the varied nature of chemical data, it is not trivial to determine the appropriate way to represent molecules. One of the earliest applications of ML techniques to spectral data was in the early 1970s, pioneered by Jurs,^22–24^ which built on classical physical organic chemistry concepts. These rudimentary ML techniques include linear regression (LR) and Fourier transform (FT), which were often converted into sequential tables and images (Figure 2). Molecular information was typically represented by fixed-length numerical descriptors, denoting physical, chemical, or topological properties. These descriptors can be derived from experimental observation such as peaks or signals in spectra, or from the molecular structure such as fingerprints, were initially used to uniquely identify molecules, compare molecular structure, and conduct similarity searching.^25^ In the decades that followed into the 1990s and 2000s, ML methods for analyzing spectral data include Principal Component Analysis (PCA) and classical statistics or Support Vector Machines (SVM) and kernel methods.^26^ In the 2010s, molecular fingerprints were converted into grid-like data such as images and processed by CNNs; these were used to identify spatial patterns and extract features for property predictions.^16,17,27^ In the 2020s, advanced transformers, multimodality, and self-supervision models started to appear.

**Figure 2:**
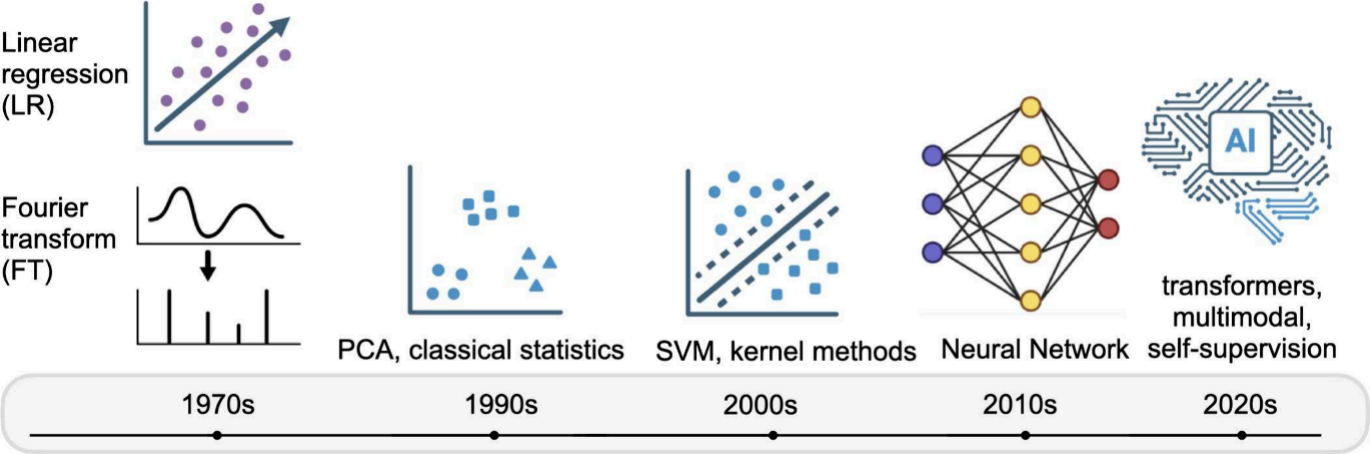
Condensed historical overview of ML.

#### 2.1.1 Molecular Fingerprints and Descriptors

A key factor of ML’s success in computational modeling lies in its capacity to contextualize molecular descriptors. There are several broad categorizations for types of fingerprints based on creation strategy, which we highlight along with methods for each category in Figure 3. The first category includes dictionary-based strategies which project molecular structure into counting statistics over a predefined set of fragments or rules,^28–35^ resulting in a discrete vector fingerprint for any molecular input with direct interpretability. Further categories broaden the space of possible features by algorithmically aggregating information over the molecular graph or 3D structure. Fragment-based methods build a more complete representation than dictionary-based approaches from the combination of all local fragments within a molecule; these methods can be split into two distinct families based on whether they aggregate path-based fragments^36–42^ or radial neighborhood fragments.^25,43–49^ For situations where the larger-scale global graph structure of the molecule is important, topology-based fingerprinting^38,50–54^ pools pairwise interaction functions weighted by distance over a molecular graph. Pharmacophore fingerprints emphasize the 3D arrangements of molecular features, finding use in prediction of protein–ligand interactions.^55–65^ A further category focuses on overall molecular shape or electrostatics to improve ligand-based screening.^44,66–69^ Finally, to vectorize chemical reactions, several reaction fingerprints have been proposed.^70–72^ Across these categories, fingerprinting methods may trade feature index interpretability for representation compactness by introducing vector hashing.

**Figure 3:**
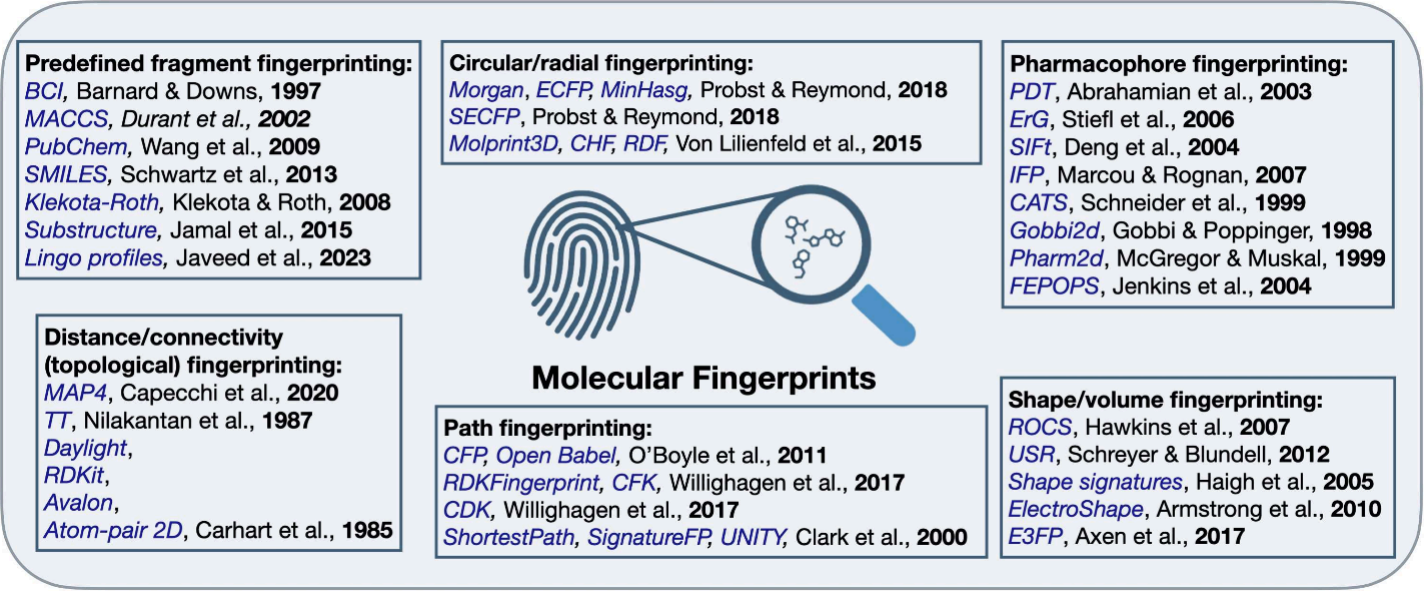
Molecular fingerprints and descriptors.

#### 2.1.2 Line Notations and String Representations

Representations of complex molecules using line notation, such as the Wiswesser line notation (WLN), have existed since the late 1940s^73^ (Figure 4). This notation was used extensively in the 1960s and 1970s due to its compactness and accessibility to new learners. In 1988, the Simplified Molecular Input Line Entry System (SMILES) was created and was widely adopted because of its ability to more explicitly describe chemical structures and compatibility with computer systems.^74^ The canonization of SMILES allowed SMILES strings to be used as unique identifiers for databases, such as PubChem and ChEMBL. However, this system was rarely used as direct input into machine learning models until the mid-2010s.^75^ Classic models for processing textual data include recurrent neural networks (RNNs)^76^ and LSTMs,^77^ but the transformer architecture popularized by BERT^78^ demonstrates the value of models designed specifically for SMILES, including ChemBERTa^79^ and MolBERT.^80^ Other systems were developed for specific use cases or to address the inherent limitations of the SMILES. A summary of the common variants is presented in Table 1. For example, InChI^81^ and Inchified/Universal SMILES^82^ are used for canonical generation, whereas SELFIES^83^ and DeepSMILES^84^ are used for robustness and validity in generation using different approaches. DeepSMILES replaces all paired syntax for ring closures and branching with a one-sided syntax encoding the size of the feature. SELFIES introduces a grammar forcing strings to be valid molecules; this increases sequence length but results in a closer relationship between changes to the string and molecule. For applications to macromolecules and polymers, BigSMILES^85^ and pSMILES^86^ were created. SMARTS strings are used for substructure searching.^87^ CXSMILES and CXSMARTS allow for the storage of external and special molecular features after a SMILES/SMARTS string.^88^ SMIRKS is used for modeling reactions.^89^ CSMILES represents 3D conformation details through 1D line notation.^90^ To improve the tokenization scheme for SMILES, SMILESPE,^91^ atom-pair encoder (APE),^92^ and hybrid fragment-SMILES tokenization (HFST)^93^ were created. In contrast to the previous tokenization schemes that increase the vocabulary size by clustering common token subsequences into a single token, SMI+AIS increases vocabulary size by differentiating common elements with different local environments.^94^

**Table 1:** Landscape of SMILES String Variants

Category	System	Purpose	Trade-off
Canonical IDs	Universal/Inchified SMILES^82^	String standardization	Requires translation
Generation/ML	SELFIES^83^	Token grammar, validity guarantee	Longer strings
	DeepSMILES^84^	Simplified rings/branches	Asymmetric notation
Queries/Annotations	SMARTS^87^	Substructure query patterns	Strict expansion of SMILES grammar
	CXSMILES/CXSMARTS^88^	Postfix special identifiers	Not an identifier
Reactions	SMIRKS^89^	Reaction transforms with atom maps	Not full mechanisms/stoichiometry
Polymers	BigSMILES^85^	Represent stochastic polymers as base units	Many polymers have same identifier
	pSMILES^86^	Polymer strings for ML	No reaction rules
3D detail	CSMILES^90^	3D conformer info encoded in 1D	Extra storage
Tokenization (ML)	SMILESPE^91^	BPE-style subwords for SMILES	Added complexity, preprocessing only
	APE^92^	Neighbor-focused tokenization	Added complexity, preprocessing only
	HFST^93^	Fragment-focused tokenization	Added complexity, preprocessing only
	SMI+AIS^94^	Differentiate element tokens by neighborhood	Added complexity, preprocessing only

**Figure 4:**
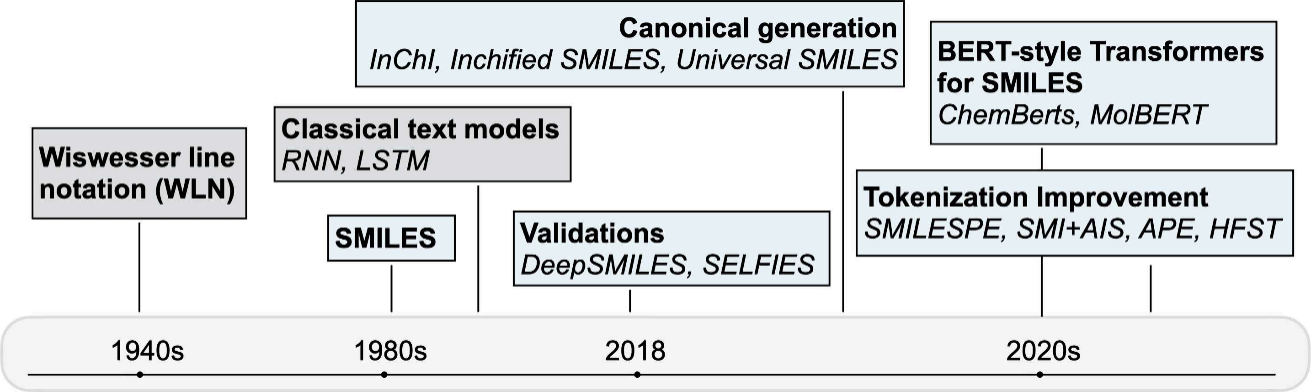
Line notations for ML models.

#### 2.1.3 Graph-Based Representations

One of the most significant limitations of line notation representations is that molecules are not 1D objects. For example, adjacency between atoms is not necessarily represented in the 1D representations. Graphs, a data format characterized by nodes and edges, are a logical way of representing molecules, using nodes as atoms and edges as bonds (Figure 5). The representation of molecules as graphs was introduced as early as the 1870s by the mathematician Arthur Cayley^95^ and has been used in machine learning systems to make predictions as early as 1999.^96,97^ With the development of classic graph neural networks (GNNs) such as GCNs,^98^ GraphSAGE,^99^ and GINs^100^ in the late 2010s, the application of GNNs to molecular data became prominent. The transformer architecture originally used for text was also applied to heterogeneous^101,102^ and homogeneous^103^ graphs around the turn of the decade, providing an additional framework for processing molecular-graph data. To explicitly model graphs as data that can be processed by computers, there are two strategies. Graphs can be represented as adjacency matrices, where each entry corresponding to the *i*th row and *j*th column determines the existence of a connection between the node *i* and *j*. The other method is an adjacency list, where all edges are represented as tuples containing the index of both connected nodes. For an edge connecting nodes *i* and *j*, the adjacency list contains the entry (*i*, *j*). An adjacency matrix is usually preferable for small graphs due to its easy implementation as a data object, whereas the adjacency list is preferable for large graphs due to its reduced memory consumption. Alongside the adjacency information, there is a feature matrix to hold information about the nodes and edges in the graph. Commonly, the features may include atom/bond type, chirality, degree,^104^ lone pairs, interactions, and bond orbitals.^105^ Because of the seemingly trivial translation from molecules to graphs, there are not as many representation variations as with line notations. However, variants do exist, such as molecular junction trees^106^ and motif graphs,^107^ which assign a full molecular substructure to a single node. A knowledge graph may be employed when interactions between chemical objects and entities besides atoms need to be modeled.^108^

**Figure 5:**
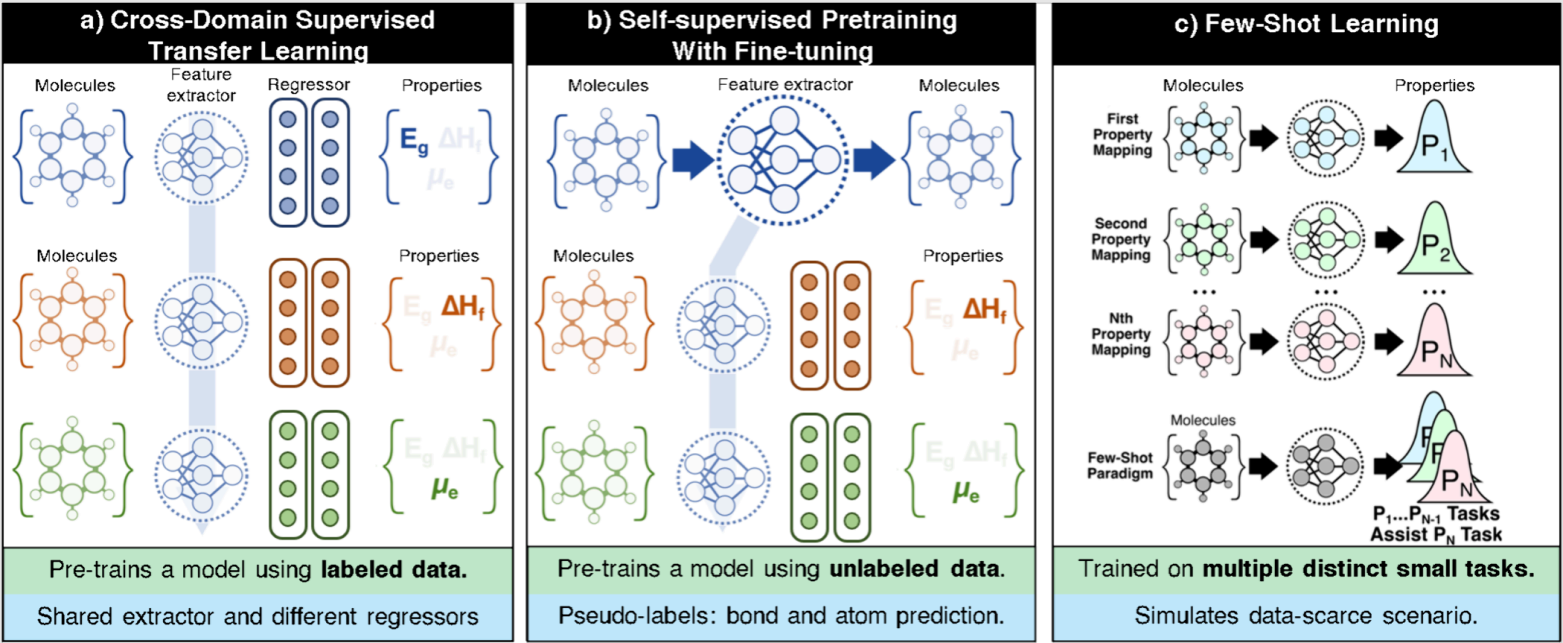
Methods to address data-scarcity in chemical modeling: (a) Cross-Domain Transfer Learning leverages knowledge from a related, abundant, labeled source task to inform a data-scarce target task. A single, shared feature extractor is pretrained on the source data, which is then reused to feed task-specific regressors for the data-scarce target tasks. (b) Self-Supervised Learning with Fine-Tuning is a two-stage process that first generates pseudolabels (e.g., bond and atom prediction) from abundant, readily available unlabeled data to pretrain a robust feature extractor. This pretrained model is then fine-tuned using the limited labeled data available for the target property. (c) Few-Shot Learning (FSL) utilizes episodic training on multiple distinct small tasks (*P*_1_ to *P*_*N*–1_) to explicitly simulate the data-scarce scenario. The goal is not just to learn the properties, but to learn a meta-learning paradigm that enables the model to adapt rapidly and generalize to a new, unseen target task (*P*_*N*_) with only a handful of examples per class.

#### 2.1.4 3D Geometric Representations

While 2D graphs capture structural connectivity, 3D geometries can provide additional information, such as atomic coordinates, interatomic distances, and angles, that may be closely linked to different chemical properties. The earliest representations of molecules using geometric information were created in the 1990s,^109^ with early machine learning applications appearing in the mid 2010s.^110–112^ It was not until models such as SchNet^113^ in 2017, DimeNet^114^ in 2020, and EGNN^115^ in 2021 that processing 3D graphs became more widely used. To generate 3D data, it is first necessary to use tools such as RDKit to generate conformations, which dictate the location of atoms in 3D coordinate space.^2^ Since then, other features included by 3D molecular representations include charge,^116^ spin,^116^ 2D atomic coordinates,^117^ Coulomb matrices,^118^ ionization,^118^ and bond order.^118^

#### 2.1.5 Emerging Multimodal Approaches

The development of models to extract molecular structure representations from the chemical literature is essential for the curation of structured data suitable for AI applications. Early work on molecule segmentation appeared in 2009 ^119^ and utilized machine vision methods. Current models improve upon this concept by utilizing a Mask R-CNN or Vision Transformer network to conduct molecule identification, masking, and segmentation.^120–122^ Once segmented, molecular images need to be transformed into one of the aforementioned representations: most commonly, SMILES. Neural models which implement SMILES-based molecular graph construction in a heuristic manner were initially used for this translation.^120,124^ However, image-to-graph encoder-decoder architectures utilizing vision transformer models have recently been developed and provide a more robust performance in 2D molecular graph generation.^125,126^

With the rise of general-use large language models (LLMs) such as GPT,^127^ their application to chemical data was inevitable. By performing domain-specific pretraining and instruction tuning on large-scale chemistry data, researchers are able to create chemistry models that are better able to reason on chemistry tasks.^128–130^ LLMs augmented with tools are able to provide additional textual features.^1,131,132^ Conversely, LLMs themselves can be used as analysis tools.^133^ Due to the limitation of text to represent the structural features of molecules, Token-Mol translates 2D and 3D features into tokens.^134^ Specific details for chemistry reasoning LLMs will be discussed in later sections.

In addition to the standard formats listed above, other unconventional ways of representing molecular data include video,^135^ structure images,^136^ and hand-drawn images.^137^ Dependency trees represent the relationships between chemical objects and enable inference through logical extrapolation.^138^ Situation calculus similarly models chemical interactions using logical deductions in PROLOG.^139^ To create an easily extensible, query-friendly representation, the XML-based format chemotypes was created.^140^ As can be seen from the examples listed above, the choice of representation for molecules is part of an ever-evolving conversation between available data and technologies. These multiple modalities pose challenges to AI algorithms, as they each carry some information about the molecule, and collectively they can help piece the molecular puzzle together. The AI algorithms not only must deal with the unique challenges in each of these modalities but also tackle the challenges that come with integration.

Having surveyed the landscape and representation of chemical data, we now turn to two complementary solution strategies that address the fundamental challenge of limited and imbalanced chemical data. Section 3 presents learning paradigms that extract maximum value from small labeled data sets through transfer learning, self-supervised pretraining, few-shot learning, and foundation models. Section 4 addresses data limitations through generation and rebalancing: synthetic augmentation expands data sets, while imbalanced regression techniques ensure models remain sensitive to rare but scientifically valuable property regions. Together, these approaches—smart learning algorithms and strategic data generation—provide a methodological foundation for robust chemical modeling under realistic data constraints.

### 2.2 Chemistry Pushes AI Boundaries

The unique data landscape of chemistry has served as a methodological forcing function for AI research. Unlike domains where data arrive in uniform formats (images, text, tabular records), chemistry demands that models reason across fundamentally heterogeneous modalities—discrete symbolic notations (SMILES), continuous geometric embeddings (3D conformers), structured relational graphs (molecular connectivity), spectroscopic signals, and natural language descriptions. This heterogeneity cannot be addressed by naive concatenation or simple feature fusion; it requires principled multimodal architectures capable of aligning representations across modalities while preserving domain-specific inductive biases. The requirement to integrate symbolic, structural, and spectroscopic information within a unified predictive framework has driven advances in cross-modal pretraining, attention-based fusion mechanisms, and domain-adaptive tokenization strategies that extend well beyond chemistry into broader scientific and multimodal AI research. Chemistry’s representational diversity thus challenges AI to move beyond single-modality methods and develop generalizable frameworks for heterogeneous data integration.

## 3 Learning with Limited Data

Building on the data landscape described in section 2, the development and application of quantitative models in organic chemistry are frequently challenged by the scarcity of labeled experimental data. Data collected in academic and industrial settings are typically sparse, whether by design or necessity. This scarcity fundamentally arises from the practical constraints of chemical research, including high cost, demanding resources, and the considerable experimental burden associated with measuring diverse sets of reaction conditions and outputs.^141^

Rather than generating synthetic data to expand data sets (section 4), the learning paradigms presented in this section maximize the value extracted from existing labeled data through knowledge transfer, unsupervised pretraining, and meta-learning.

As limited labeled data is not unique to chemistry, several established computational learning paradigms shown in Figure 6 have been adapted to the domain to address this challenge:

- **Cross-Domain Supervised Transfer Learning**, a methodology where a model is initially trained on a large, fully labeled source domain and the learned general knowledge (e.g., feature extractors, representations) is then transferred and adapted to a distinct but related target domain to efficiently solve a task with only a limited number of target labels.
- **Self-Supervised Pretraining with Fine-tuning**, A two-stage process where *Pretraining* uses unlabeled data and self-generated “pseudolabels” (e.g., predicting masked-out parts, contrasting different views) to learn robust, general-purpose feature representations, followed by *Fine-tuning*, which adapts these representations using the small amount of task-specific labeled data.
- **Few-Shot Learning**, where the model is explicitly trained to *learn how to learn* from minimal data (also known as meta-learning). Training proceeds in “episodes” or “tasks,” and the objective is to optimize the model’s initialization or learning algorithm such that it can solve a new, unseen task by observing only a few support examples (the “shot”) and achieving high accuracy on the associated query examples.

**Figure 6:**
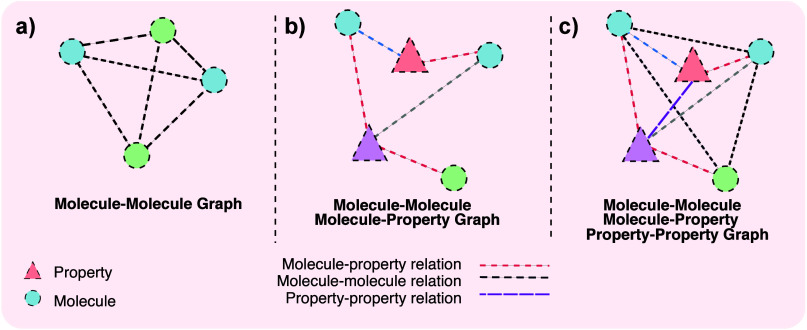
Hierarchy of relational learning for chemical data. This figure illustrates the progression from (a) modeling molecule–molecule similarities to (b) the development of molecule–property relational graphs, and finally (c) the exploitation of property–property relationships (GS-Meta/KRGTS) to address the challenges of task-specific data scarcity in organic chemistry.

Each of these strategies embodies a different philosophy for improving the data efficiency. The following subsections describe how they are implemented and evaluated in chemical modeling, beginning with cross-domain supervised-transfer learning.

### 3.1 Cross-Domain Supervised Transfer Learning

Cross-Domain Supervised Transfer Learning mitigates data sparsity by reusing knowledge from one chemical domain or task to improve performance on another related task with limited data.^142^ A model is first trained on a large, accessible data set (the *source task*), which may consist of low-fidelity simulations^143^ or broad molecular collections with simpler annotations. The learned knowledge, such as general molecular representations or shared structural features, is then transferred to address another high-fidelity task (the *target task*) by fine-tuning the model trained in the source domain on a small data set in the target domain.^144^

This procedure typically separates the model into two components: a *feature extractor* that captures transferable representations and one or more *task-specific heads* that adapt these representations to downstream objectives.^144,145^ Such hierarchical architectures allow the model to preserve general chemical knowledge while being specialized to new prediction targets.

Transfer learning has been successfully applied across diverse chemical modeling problems, including molecular property prediction,^143,146^ reaction prediction,^147,148^ and Retrosynthesis Prediction.^149–151^ For example, pretrained Transformer or graph neural network models trained on large reaction corpora (e.g., USPTO) can be fine-tuned on smaller data sets for specific reaction types, achieving improved generalization in low-data regimes.^152–156^ This approach is particularly valuable when mechanistic similarity exists between the source and target domains, enabling efficient transfer of learned representations and accelerating convergence on scarce, high-value chemical data.^156^

### 3.2 Self-Supervised Pretraining with Fine-Tuning

The concept of Cross-Domain Transfer Learning narrows the definition to transferring knowledge from related source tasks trained in a supervised manner to improve learning on a target task with limited data. In contrast, Self-Supervised Learning (SSL) approaches typically rely on large-scale unsupervised pretraining followed by supervised fine-tuning adaptation on a specific target task. By generating pseudolabels from the intrinsic molecular structure, SSL eliminates the need for molecule-level annotations and enables the use of large-scale data sets.^157–159^ These pseudolabels allow the model to capture meaningful molecular features, which can then be fine-tuned on limited labeled data for specific downstream tasks, such as solubility, toxicity, or biological activity prediction.

A common family of SSL approaches relies on *masking or reconstruction* objectives.^160,161^ In molecular graph representations, models are trained to predict masked atomic or bond features, thereby learning local chemical context.^159,162^ In text-based representations such as SMILES or SELFIES, autoregressive or masked-token prediction objectives are used to reconstruct corrupted molecular strings, analogous to masked language modeling in natural language processing (NLP).^163^ These methods enable the model to capture both atom-level and substructure-level dependencies in a data-efficient manner.^79,164^

Complementary to generative learning, *contrastive* SSL methods have also been developed.^165–167^ Contrastive learning frameworks, such as GraphCL^168^ and MolCLR,^169^ maximize agreement between different augmentations of the same molecule while distinguishing it from others, due to robust and invariant representations.

Overall, SSL serves as a powerful foundation for learning transferable molecular representations without manual labeling. By exploiting structural regularities within unlabeled data, SSL complements transfer learning and enables downstream fine-tuning even in severely label-scarce regimes^152,162^ and has shown promising performance in reaction prediction.^156^

### 3.3 Few-Shot Learning

The training paradigm of few-shot learning (FSL) differs fundamentally from conventional transfer learning or pretraining with fine-tuning. Few-shot learning trains models in an episodic manner, simulating many tasks where only a few labeled examples per class are available to develop the ability to rapidly adapt to unseen tasks.^170^

The foundational step in applying FSL is the construction of a standard supervised data set into a meta-learning episodic paradigm, which is crucial because it simulates the real-world data scarcity challenges.^171^ Instead of a single, large training set, the data is algorithmically partitioned into a series of independent, miniature tasks, each a self-contained few-shot problem defined by a **support set** and a **query set.** ^170^ For classification problems, the model is trained to determine if a new compound corresponds to a specific class (e.g., “active” or “inactive”) based on a limited number of known examples.^170^ The support set provides a limited number of labeled examples per class (referred to as an *N*-way *K*-shot problem) for rapid task learning, while the remaining examples from those same classes are designated as the query set, upon which performance is evaluated.^170,172^ Training across numerous such tasks compels the model to develop a generalized ability to “learn how to learn” from minimal data, ensuring performance gains are a result of robust generalization capabilities and not just overfitting to a particular data set.^172,173^

### 3.4 Property Prediction with Limited Data

Since the scarcity of labeled molecular data poses significant challenges for molecular property prediction, a wide range of approaches has been proposed to leverage FSL to enhance performance under limited supervision. Metric-based approaches constitute a prominent category of FSL models; these methods learn an embedding space where molecules are transformed into vector representations, such that compounds with similar properties or structures are positioned in close proximity. When a new task arises, the model classifies a new compound by comparing its embedding to the limited examples in the support set.^172,173^ Certain architectures inspired by relation networks combine individual molecule embeddings with class-specific representations to compute similarity scores.^174,175^ For instance, the hierarchically structured learning model predicts molecular properties by constructing a global relation graph based on structural similarities, which facilitates the transfer of knowledge across related tasks.^176^ Other implementations, such as siamese networks, reinforce the distinction between training examples by minimizing the distance between similar pairs while maximizing it for dissimilar ones.^177,178^

In gradient-based approaches, the focus is on identifying an optimal initialization point for model parameters, allowing for rapid adaptation to new tasks through minimal gradient updates.^179^ This strategy is effective for fine-tuning models on small data sets, as demonstrated by the MetaGNN framework. On the basis of the model-agnostic meta-learning (MAML) algorithm, MetaGNN integrates a task-attention module to weight the importance of specific training episodes.^179,180^ To further mitigate data scarcity, these models often incorporate self-supervised modules that leverage unlabeled data via auxiliary tasks such as bond reconstruction and atom type prediction.^180^ Subsequent developments have integrated graph neural network embeddings with traditional molecular fingerprints to capture both learned and expert-defined structural features, thereby increasing the robustness of the resulting property predictions.^181,182^

Recent frameworks have increasingly adopted hierarchical matching and parameter-efficient strategies to address task heterogeneity. The UniMatch framework combines a hierarchical matching mechanism with task-level meta-learning to capture features across multiple molecular scales.^183^ Similarly, the ProtoMAML method integrates the inductive biases of Prototypical Networks with the adaptive capabilities of MAML to improve parameter initialization.^182^ To avoid the computational cost of full-model updates, Pin-Tuning employs lightweight adapters and Bayesian weight consolidation, which facilitates parameter-efficient tuning while preventing the catastrophic forgetting of pretrained knowledge.^184^ These structural priors are further refined in the Pre-PAR+MTA model, which recursively decomposes molecules to identify frequently occurring motifs.^185^ By treating these motifs as representative building blocks, the model establishes a more consistent feature space for downstream meta-learning tasks.

Further architectural developments have combined graph-based, convolutional and attention components to capture local and global dependencies within molecular graph embeddings.^186–190^ Models like EM3P2,^191^ AttGNN^192^ and PH-Mol^193^ integrate other aspects such as uncertainty quantification, pretrained and fine-tuned models as priors, and property descriptions as vector representations. AR-APM^194^ uses an active learning process to create pseudolabels to improve performance.

Conventional few-shot molecular property prediction relies on one-to-one mappings between individual molecules and specific properties.^195–197^ This paradigm focuses primarily on molecule–molecule relationships, as illustrated in Figure 6a, but it frequently overlooks the many-to-many correlations inherent in multiproperty data sets. Crucially, these models fail to account for the latent relationships that exist between different properties themselves. Advanced FSL models address this limitation by adopting a relational modeling approach that incorporates property-level dependencies.^176,198,199^

The graph sampling-based meta-learning (GS-Meta) framework implements this relational strategy by constructing a molecule–property relation graph (MPRG). In this structure, molecules and property nodes are connected by edges defined by property labels (Figure 6b). To address the episode dependency inherent in these intersecting graphs, GS-Meta utilizes a contrastive loss function to schedule the subgraph sampling process. This mechanism ensures that subgraphs corresponding to the same target property maintain an internal consistency while remaining distinct from those associated with different properties.^198^

These relational concepts are further extended by models designed to manage task heterogeneity through disentanglement and multiplexing. Meta-DREAM employs a disentangled graph encoder and a soft clustering module to isolate the underlying factors that govern different property clusters.^200^ This enables the customization of knowledge transfer based on the specific characteristics of each task group. Similarly, the KRGTS framework expands the MPRG concept into a Molecule-Property Multi-Relation Graph by incorporating multiplex graph views based on molecular scaffolds and functional groups.^199^ A learnable sampler is then used to retrieve auxiliary properties that share significant relationships with the target property, thereby enhancing the model’s contextual awareness (Figure 6c).

Few-shot principles have also been extended to regression tasks, where models predict continuous molecular properties rather than discrete categories. Deep kernel Gaussian processes (DKGPs) address these problems by integrating meta-learning with deep kernel learning.^201^ This integration is particularly effective because the Gaussian process prior provides a formal probabilistic framework that regularizes the model, thus preventing overfitting in data-scarce regimes. By combining the flexibility of deep neural networks with the robust uncertainty quantification of Bayesian methods, DKGPs avoid the rigid assumptions often associated with traditional meta-learning architectures.

While few-shot methods demonstrate significant potential for learning from limited examples, their performance remains highly sensitive to data quality and distribution. Early evaluations relied on classical benchmarks such as Tox21 and PCBA, yet these data sets often introduce biases through fixed task splits and simulated scarcity.^202^ Consequently, research has shifted toward purpose-built platforms like FS-Mol, which are designed to replicate the imbalanced and sparse data characteristic of real-world drug discovery.^203^ FS-Mol strictly separates training and testing tasks to ensure rigorous evaluation of generalization. Furthermore, the choice of evaluation metrics has evolved to reflect these data challenges; for instance, while the Area Under the Receiver Operating Characteristic Curve (AUC-ROC) remains a standard for ranking, the Delta Area Under the Precision-Recall Curve (ΔAU-PRC, measuring the performance change in AUPRC) is increasingly preferred for highly imbalanced molecular data sets.^202^

### 3.5 Reaction Prediction with Limited Data

Few-shot reaction prediction refers to the task of predicting reaction outcomes for new reaction types when only a handful of labeled examples are available. Unlike conventional approaches that rely on large-scale data sets, it emphasizes rapid adaptation and generalization across tasks, often leveraging meta-learning or transfer learning frameworks. This setting reflects the data-scarcity challenge in chemistry and provides a pathway toward models that capture fundamental reaction principles under low-resource conditions. For example, a pretrained Transformer model was fine-tuned on 80% of 9,959 Heck reactions,^153^ demonstrating the concept of knowledge transfer, as the fine-tuned model showed improved prediction accuracy on Heck reactions compared to the pretrained model. Another pretrained model was fine-tuned on 80% of 2,254 Baeyer–Villiger reactions,^154^ enabling the model to handle this reaction type, which was unseen during pretraining. Similarly, fine-tuning was performed on 200 samples of the Suzuki reaction and 148 samples of Barton’s bismuth arylation reaction to improve prediction on a similar reaction type,^155^ the Chan-Lam coupling reaction. Meta-learning was leveraged to enable reaction prediction with rapid adaptation to unseen reaction types using only a few training samples^204^ (using only less than 5 samples). Transfer learning with chemically related reactions has also been introduced to improve prediction in low-data regimes by leveraging mechanistic similarity between reactions.^156^ Bayesian meta-learning has been introduced in few-shot reaction prediction domain^205^ for binary classification of asymmetric hydrogenation of olefins (AHO) outcomes, and this method focuses on selectivity classification within the AHO domain. Few-shot retrosynthesis is another challenging problem, which is elaborated in detail in section 5.1.

### 3.6 In-Context Few-Shot Learning with Foundation Models

Foundation models typically exist as base models, which are often developed through unsupervised/self-supervised pretraining on a large amount of unlabeled data.^206^ Unlike traditional models that require retraining for specific task, foundation models enable adaptation through fine-tuning or prompting, allowing knowledge reuse across diverse downstream tasks. A key mechanism underpinning this adaptability is in-context learning (ICL),^207^ in which a model learns to infer tasks and make predictions directly from examples or instructions provided in its input context without updating its parameters. Depending on whether a few or no demonstrations are given, this process manifests as few-shot or zero-shot learning.

Large language models (LLMs) such as OpenAI GPT models,^208^ Google Gemini,^209^ or Llama models^210^ exhibit strong in-context learning abilities when provided with well-crafted demonstrations and prompts,^211,212^ making them the most popular generalized foundation models. This paradigm marks a fundamental shift from parameter-based learning to context-based learning, representing a new mechanism for data-efficient learning in chemistry and other scientific fields.

This success has motivated researchers to leverage foundational models to solve scientific problems. However, the absence of domain-specific knowledge in general-purpose pretraining limits their performance in the field of chemistry,^212,213^ which leads to the development of specialized foundation models, trained on molecular, reaction, or text corpora that better reflect chemical semantics. Early efforts, such as ChemBERTa^79^ and ChemBERTa-2,^164^ focused on textual representations and pretrained on millions of molecular entries, have achieved competitive performance on MoleculeNet.^214^ Graph-based pretrained models, including GROVER^162^ and MolCLR,^169^ leveraged large unlabeled graphs to learn transferable representations, thereby improving sample efficiency and facilitating few-shot adaptation in downstream property prediction.

Recent advances have expanded the scope of chemistry foundation models toward multimodal and reasoning-oriented architectures. Models such as ChemDFM,^129^ Ether0,^130^ LLaMat,^215^ and Nach0,^216^ integrate molecular graphs, reaction text, and experimental data to enhance generalization and reasoning capabilities. In parallel, agentic frameworks demonstrate how LLMs can interact with external tools to support complex chemical scientific discovery workflows.^217–219^ Collectively, these advances extend few-shot and zero-shot learning into real chemical contexts, aligning with recent findings^220^ that foundation-model-based approaches surpass both traditional machine learning and transformer baselines in extremely data-scarce regimes.

As few-shot models become more complex and “black-box” in nature, ensuring their predictions are reliable and can be interpreted by chemists is a major challenge. Understanding the “why” behind a prediction is as important as the prediction itself, particularly for high-stakes applications like drug discovery. The ability to quantify the uncertainty of a prediction is also critical for knowing when a model’s output can be trusted, especially when predicting properties for compounds that are far from the training data.

Complementary to these learning paradigms, data augmentation strategies that synthetically expand training sets through generation in feature, latent, or structure spaces are discussed comprehensively in section 4.2.

### 3.7 Chemistry Pushes AI Boundaries

Chemistry’s fundamental data-scarcity problem, arising from the combinatorial explosion of chemical space, the high cost of experimental measurements, and the systematic underreporting of negative results, has pushed algorithmic innovation in learning from limited supervision. While computer vision and natural language processing benefit from massive labeled corpora (millions to billions of examples), reaction and property prediction in organic chemistry routinely operate with hundreds to thousands of measurements per task, and novel reaction families or rare functional groups may have fewer than ten recorded instances. This extreme scarcity has driven the development and refinement of transfer learning strategies (pretraining on broad chemical corpora before task-specific finetuning), self-supervised pretraining objectives tailored to molecular and reaction representations, meta-learning frameworks that explicitly optimize for rapid adaptation from few examples, and foundation models that distill generalizable chemical knowledge from heterogeneous data sources. These paradigms, now widely adopted across scientific machine learning and low-resource domains, originated in part from the necessity of extracting reliable predictions from chemistry’s sparse and imbalanced data landscape.

## 4 Data Rebalancing and Generation

Section 2 outlines how sparse measurements, long-tailed label distributions, and heterogeneous modalities undermine chemical ML pipelines. While section 3 addressed limited labeled data through smart learning paradigms, this section takes a complementary approach: generating synthetic training data and developing algorithms that remain sensitive to rare but high-information regions of the property space. The molecular properties chemists value most (e.g., potent binders, extreme transport coefficients, high-yield reactions) are rare in experimental data sets. These exceptional cases appear infrequently, and collecting enough examples through additional experiments is prohibitively expensive. Even sophisticated descriptors cannot overcome this fundamental problem: models underperform in regions of the property space most relevant for predictions because they rarely see examples from those regimes during training.

This challenge has been addressed via two complementary approaches, which we describe below. First, we survey data augmentation and generation strategies across feature, latent, and structural spaces. These allow the expansion of data sets and enrich underrepresented property regions without requiring expensive new experiments. Second, we examine methodological advances for imbalanced regression, from relevance-aware losses to chemistry-specific graph augmentations that maintain model sensitivity to tail events, even when synthetic data generation is insufficient. Together, these methods provide practical tools for improving model performance on limited and imbalanced chemical data sets before applying them to downstream tasks such as reaction prediction or reasoning.

### 4.1 Imbalanced Regression

Molecular data sets in chemistry are frequently *imbalanced* for both classification and regression tasks. While class imbalance has been widely explored, *imbalanced regression* remains comparatively underdeveloped, especially for molecular property prediction, where the targets are continuous and often long-tailed.^221^

#### 4.1.1 Data Scarcity and Extreme Value Prediction

Many core predictive tasks in chemistry (e.g., molecular solubility, reaction yield, binding affinity, and bioactivity) require modeling continuous properties that are unevenly distributed across the label space.^221^ This imbalance often leads to systematically poorer performance in the most interesting regions of the target space: the rare and extreme values. In virtual screening and molecular optimization, for example, the primary goal is to identify molecules with exceptional properties suitable for experimental follow-up.^222–225^ However, such compounds are typically underrepresented in data sets,^226–231^ which constrains a model’s ability to generalize beyond the dense, low-value regions and limits its overall utility for discovery-driven applications.

In *AI-driven drug discovery*, these challenges are particularly acute. Data scarcity, experimental noise, and the long-tailed nature of molecular property distributions, i.e., the portion of the distribution having many occurrences far from the head or central part of the distribution, collectively hinder regression models,^197,232^ as demonstrated in Figure 7. For enzyme kinetics, rare extreme values of parameters such as *k*_*cat*_ and *K*_*m*_ are biologically significant yet sparsely observed. Furthermore, contextual factors such as pH and temperature that are known to influence reaction dynamics are often omitted, exacerbating prediction bias. The resulting skewed empirical distributions closely resemble those of long-tailed image recognition data sets, where a small number of well-sampled categories dominate while rare categories contain only a few examples.^233–237^ Consequently, current models frequently perform poorly in the regions where accurate prediction would yield the greatest biological and chemical insight.

**Figure 7:**
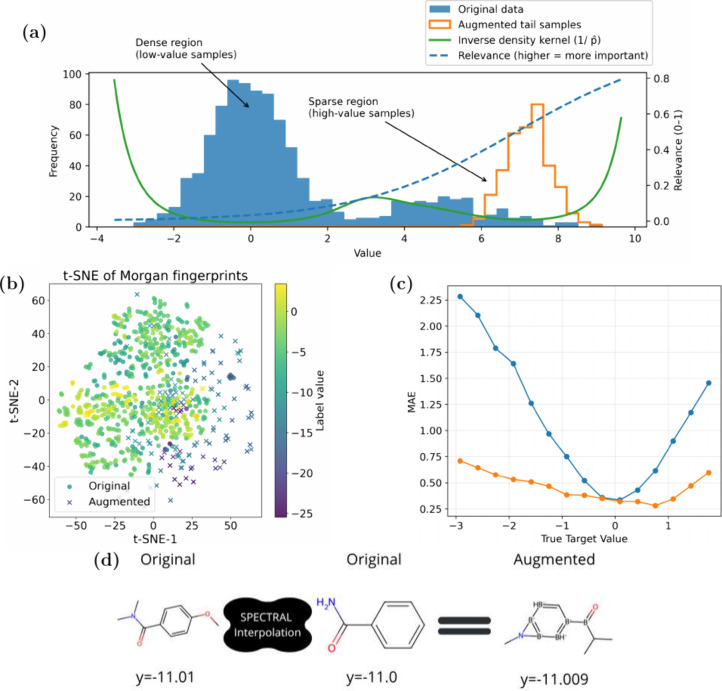
(a) Imbalanced regression distribution: most samples lie in low-value regions, while high-value compounds are rare but scientifically important. Augmentation methods (e.g., SMOGN, SMH) enrich extreme-value regions, and inverse-density kernels (e.g., LDS, BMSE) down-weight dense areas. The relevance function (e.g., SERA) emphasizes the importance of extreme targets. (b) Example of augmented molecules in underrepresented space using SPECTRA. (c) Example of performance improvement with augmented data using SPECTRA. (d) Example of SPECTRA interpolation-based augmentation.

A similar phenomenon arises in *polymer informatics*. Here, predictive models must often extrapolate beyond the dense regions of the training distribution to satisfy user-defined design specifications that lie in sparse or unseen areas of property space.^238–240^ Such distribution shifts introduce heightened uncertainty and limit the applicability of standard ML techniques that assume independent and identically distributed data. Moreover, large-scale chemical data sets employed for pretraining and transfer learning—though rich in diversity—remain highly imbalanced across molecular and reaction spaces.^241–244^ This imbalance, coupled with the sparsity of extreme cases, impedes model generalization and knowledge transfer.^163,245^ Addressing these issues requires strategies that explicitly model rare-value importance, ensuring that learning and inference remain effective in the chemically and scientifically critical extremes of the data distribution.

Ma et al.^230^ explicitly frame reaction yield prediction as imbalanced regression: yields from unoptimized reaction screens are skewed toward lower values, causing models to undervalue high-yield reactions most relevant to synthesis planning. Reweighting high-yield samples improves accuracy in these rare regions without sacrificing overall performance—illustrating that imbalance-aware regression yields more practically meaningful chemical predictions.

#### 4.1.2 Imbalanced Classification

Class imbalance is a long-standing challenge in machine learning, particularly in domains where the frequencies of samples are unevenly distributed across categories. In chemistry and related fields, this problem is often amplified because of the limited availability of experimentally validated data for certain molecular classes or properties. Classical strategies developed for *classification imbalance*, including resampling, cost-sensitive learning, and posterior recalibration, have laid the foundation for more recent adaptations to regression. However, extending these techniques to continuous targets introduces new questions: defining minority regions along a continuum and ensuring coherent sample generation or weighting across the entire target space remain nontrivial tasks.^246^

Traditional approaches to class imbalance generally fall into two categories: *data level* and *algorithm level* methods. At the data level, resampling strategies are widely used to balance class frequencies. Techniques such as random undersampling remove redundant samples from majority classes, while oversampling replicates or synthetically augments minority data. One of the most influential oversampling algorithms, SMOTE (synthetic minority oversampling technique),^247^ generates synthetic samples by interpolating between neighboring instances of the minority class, effectively enriching low-density regions of the feature space.

Beyond data resampling, *cost-sensitive learning* methods^248,249^ modify the training objective to assign higher loss weights to misclassified minority samples, compelling the model to focus more on underrepresented classes. In contrast, *posterior recalibration* techniques^250–252^ adjust predicted probabilities or decision boundaries after training, increasing the effective decision margin for minority categories. These methods collectively enhance robustness and generalization when facing highly skewed label distributions.

Recent research has extended imbalance handling into the realm of *graph learning*, where node- or graph-level classification tasks frequently exhibit severe imbalance.^253^ In molecular machine learning, similar principles have been adopted: graph neural networks (GNNs) are trained with reweighting schemes or augmented data sets to correct biases in node classification and molecular activity prediction. Furthermore, in chemistry-specific applications, deep generative models such as generative adversarial networks (GANs) and variational autoencoders (VAEs) have been leveraged to augment minority classes, producing structurally valid molecular representations and enriching underrepresented chemical subspaces.^254^

#### 4.1.3 Methodological Advances for Imbalanced Regression

A major family of approaches targets the data distribution directly, generating new samples or resampling existing ones to better represent sparse regions of the label space. SMOGN^255^ adapts SMOTE for continuous targets by synthesizing samples in low-density areas. G-SMOTE^256^ generalizes this process by sampling within geometric neighborhoods (such as hyperspheres) rather than interpolating along straight lines, increasing diversity in underrepresented ranges. Ensemble approaches such as REBAGG^257^ incorporate relevance-guided resampling into bagging, improving model exposure to rare values. More recently, WSMOTER^258^ estimates the local density of labels and generates new samples proportional to rarity, eliminating the need to define minority boundaries through arbitrary thresholds.

A second line of work focuses on modifying the training objective to emphasize important or rare regions of the label space. Focal loss^249^ increases the weight of the harder-to-predict samples. Density-aware objectives such as DenseLoss^259^ and LDS^260^ scale per-sample importance by estimated target density, reducing redundancy in dense regions, and amplifying learning on rare examples. BMSE^261^ rebalances sensitivity across the continuous target range via logit calibration. Structural regularizers such as RankSim^262^ encourage consistency between local neighborhoods in feature space and the corresponding relationships in label space, helping preserve the geometry of rare values. On the evaluation side, the SERA metric^221^ introduces a relevance-weighted error that prioritizes accuracy where domain importance is highest, aligning the assessment with practical priorities.

Beyond data and loss design, several recent methods introduce complementary learning paradigms. SIRN^263^ leverages unlabeled samples through pseudolabeling and label-anchored mixup to enrich scarce regions. ConR^264^ employs contrastive regularization to maintain correspondence between relationships in feature and label spaces, mitigating collapse around dense target values. Variational imbalanced regression^265^ incorporates conjugate priors (normal–inverse-gamma) to probabilistically reweight skewed data, improving uncertainty estimation under imbalance. Finally, IM-Context^266^ bypasses retraining entirely by retrieving minority-rich examples for in-context learning, enabling rapid adaptation to rare targets.

#### 4.1.4 Domain-Specific Adaptations in Chemistry

In chemistry, models must account for the fact that compounds with the most desirable properties—high potency, selectivity, or stability—are often scarce. Recent work adapts graph-based learning to better capture these extremes. SGIR^267^ reduces imbalance effects by combining inverse distribution, confidence-based pseudolabeling with latent-space augmentation, gradually enriching sparse regions. SMH^268^ generates new molecules directly in the spectral domain by perturbing Laplacian eigenmodes, producing structure-preserving samples in rare-value ranges. Building on these ideas, SPECTRA^269^ interpolates molecular graphs in spectral space. By aligning Laplacian components, interpolating spectral and feature information, and reconstructing topology, SPECTRA generates physically plausible molecules whose estimated properties fall in underrepresented target regions. These approaches use density- or rarity-aware sampling to direct augmentation where data are scarce, improving predictive accuracy.

Imbalance mitigation was also incorporated into task-specific molecular pipelines. Multimodal models that combine SMILES strings with 3D conformers^232^ often employ attention mechanisms^270^ and distribution-smoothing techniques such as FDS^260^ to reduce the bias toward common ranges. For enzymatic kinetics, UniKP^271^ integrates protein language models with substrate structural encodings and applies reweighting to improve the prediction of high-value kinetic parameters. In reaction modeling, the HeckLit data set^272^ demonstrates how literature-derived corpora can be leveraged to predict catalytic yields here, distribution smoothing and subset-based training help maintain accuracy for high-yield reactions, which are comparatively rare.

### 4.2 Data Augmentation/Generation Strategies

Molecular data are often small in size and imbalanced in the chemistry tasks. For example, the ChEMBL database^273^ (version 35, updated on 2024–12–01) records biological assays for over 2.5 million molecules but contains only about 21 million activity values spanning 1.7 million different biological assays.^274^ For each task, common ML benchmarks typically provide only thousands of labels, which is insufficient for training modern ML and AI models that are extremely data-hungry.^214^ Many molecular tasks are framed as regression problems in ML, where the label space can be very large, ranging from values close to 0 up to more than 10,000.^267^ An example is polymer property regression for oxygen permeability compiled in a database for polymer permeability experiments between 1950 and 2018.^275^ Examination of the label distribution shows that 88% of the values lie in the range between 0 and 100, even though the maximum value exceeds 15,000.^267,275^

To overcome the challenges of limited and imbalanced supervision in molecular machine learning, researchers have developed different data augmentation strategies. Unlike new label annotation, which requires costly and time-consuming laboratory experiments,^273,276^ data augmentation provides an efficient and inexpensive means to expand the training set during model development. Formally, a data augmentation operation can be viewed as a function that takes an existing data point (input–label pair) and produces a new one that increases data set diversity while preserving the label.^277,278^ For molecular data, these transformations are applied to structural, atomic, or bonding features under defined rules to either preserve or adjust the target property. Notably, augmented samples do not always correspond to explicit molecular structures—they can also exist as virtual representations in the neural network’s latent space. As illustrated in Figure 8, the following subsections describe data augmentation and generation in the feature, latent, and structure spaces.

**Figure 8:**
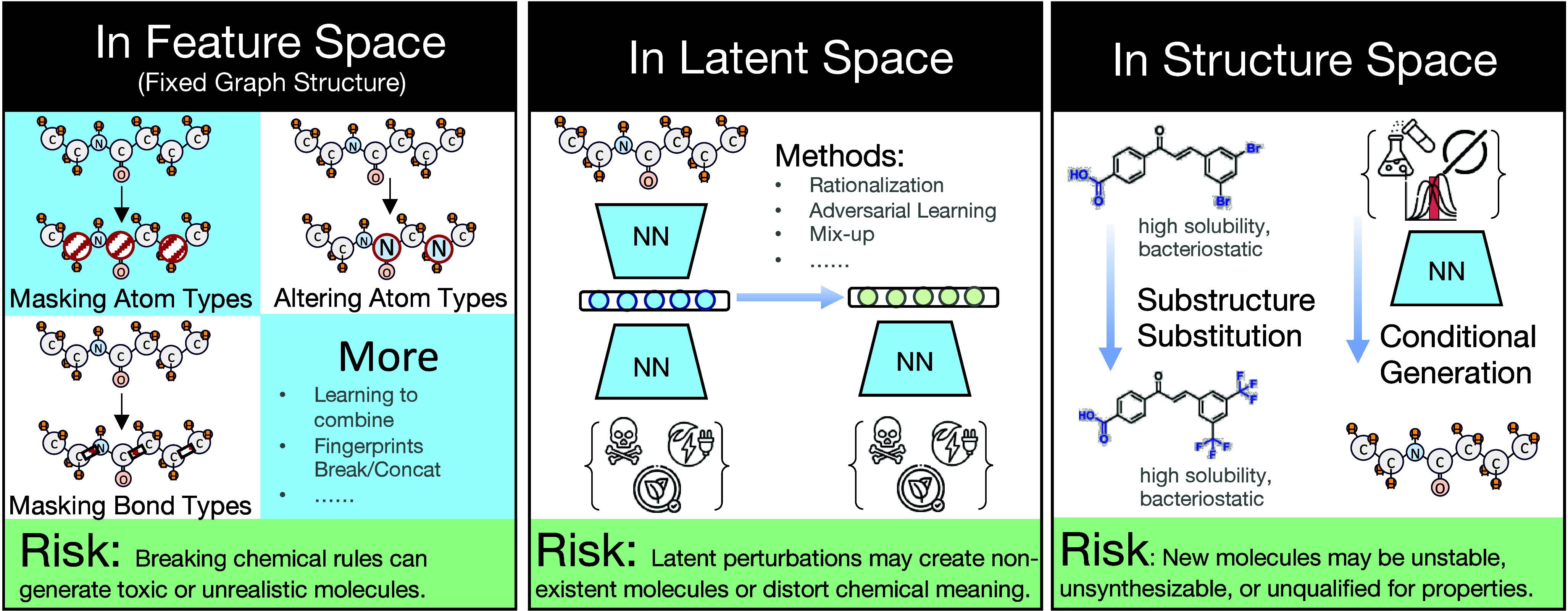
Data augmentation can operate in the feature, latent (neural network or NN), and structure spaces (Section [sec:Data augmentation]). Each method has its own advantages and risks. In the feature space, data augmentation is efficient but may break chemical rules, leading to unrealistic molecules. In the latent space, data augmentation can be optimized using various machine learning methods but risks creating virtual data points that correspond to nonexistent molecules. In the structure space, substructure substitution or generative models can be guided to create new molecules, but the generated structures may again be unstable, unsynthesizable, or not suitable for specific property requirements.

#### 4.2.1 Data Augmentation in Feature Space

Molecules have atomic and bond features that can be masked or perturbed to create fragments or analogs of the same molecule. This approach leads to smaller perturbations of the original structure and therefore increases the probability that a valid molecule is generated. Atoms possess multiple features, such as atom type, chirality, degree, and formal charge, while bonds have features such as bond type, bond order, and stereochemical information. These features can be partially or fully masked,^279^ and the masking distribution^280^ can be adjusted to control task difficulty and prevent changes in the molecular label. Furthermore, it is possible to learn to automatically select the most suitable data augmentation strategies for a given objective.^281^ For example, a predefined set of strategies, such as atom type masking, atom chirality perturbation, bond type masking, and bond stereo perturbation, can be formulated as a bilevel optimization problem instantiated as a min–max optimization:^281^ first sampling the most challenging augmentation strategies according to the current loss, and then minimizing the loss function to improve the model. Consequently, the sampling distribution over augmentation strategies differs across data sets, maximizing the benefits of diverse augmentation approaches. In addition, automated data augmentation can be used to prevent compromising label-related information in molecular classification tasks^282^ and to better preserve label consistency.

Pure feature-wise data augmentation does not consider structure-wise information, whereas chemical properties are determined not only by atoms and bonds in isolation but also by their spatial arrangements and connectivity. To address this, fingerprints such as ECFP and RDKFP^38,43^ can convert structural information to feature vectors for data augmentation. For example, Fingerprint Break^277^ leverages the Breaking Retrosynthetically Interesting Chemical Substructures (BRICS) algorithm^283^ to decompose molecular structures at multiple levels and assign the original label to the fingerprints of BRICS fragments when their Tanimoto similarity is greater than a threshold. Fingerprint concatenation, in contrast, concatenates fingerprint vectors from highly similar fragments of the original molecule to augment the training set.

##### 4.2.1.1 Risks and Future Direction

Feature-space data augmentation typically preserves molecular structure to maintain label consistency and is computationally efficient for enlarging data sets and reducing overfitting. However, its effectiveness is highly task-dependent and requires careful design, as inappropriate perturbations can break chemical rules and introduce noise examples. For instance, altering bond features such as stereochemistry may invert the chiral configuration of thalidomide between its (*R*) and (*S*) forms, transforming a therapeutic compound into a teratogenic one. This illustrates the fact that small perturbation of the structure can have large effects in the property and in this case a safety risk: random feature-space augmentations can yield toxic or unrealistic molecules, compromising model reliability and real-world applicability. Future work should incorporate constraints such as (stereo)­chemical validity to ensure more reliable molecular augmentations and more stringent check on the validity and synthesizability of generated structures. Even then, the benefits of data augmentation need to be balanced against the risk that the hypothetical, generated molecules could have very different properties compared to their progenitors even for minor perturbations, as is the case for discontinuous “activity cliffs” in medicinal chemistry.

#### 4.2.2 Data Augmentation in Latent Space

Data augmentation can generate virtual molecular data directly in the latent space by creating new representation vectors without decoding them to explicit molecular structures. This approach avoids the production of chemically invalid molecules while providing additional efficiency and flexibility. Graph neural networks (GNNs) can encode atomic, bonding, and structural information into latent representations, where new data points can be dynamically created during training. For example, in graph-rationalization methods,^284,285^ GREA^285^ uses a separator GNN to output a vector denoting the roles of each atom in the latent space. The atom could be either within a rationale or an environment. A rationale is a molecular substructure that supports the model predictions. The environment, which complements but is not causally related to the label, is collected within a training batch. New data are created by combining the rationale with different environments directly in the latent space.

Latent space augmentation enables techniques such as adversarial learning^286^ and imbalanced learning.^267^ Adversarial training formulates a min–max objective that maximizes perturbations in latent representations while minimizing prediction loss. In imbalanced learning, SGIR^267^ divides the label space into intervals, aggregates representations within each interval as anchors, and mixes them with training representations to generate new samples. This label-anchored mixup directs augmentation toward underrepresented regions of the label space, mitigating bias. Another latent-space strategy, mixup,^287^ forms convex combinations of two feature–label pairs. Adaptations for molecular graphs^288^ mix node-level features through message passing and then combine graph-level representations. Mixup assumes that linear interpolations in latent space correspond to smooth transitions in target space, a principle also used in oversampling methods such as SMOTE.^247^

##### 4.2.2.1 Risks and Future Direction

Data augmentation in the latent space introduces new risks related to the semantics of the latent representations. The latent space learned by neural networks does not always preserve chemical continuity or physical constraints; therefore, the assumptions behind many augmentation approaches require careful consideration. For example, linear interpolation may produce representations that correspond to nonexistent molecules when decoded. Adversarial perturbations can push latent vectors beyond the domain of chemically meaningful representations, leading to misleading gradients or overfitted models that exploit artifacts rather than true chemical relationships. In rationale-based augmentation, incorrect separation between the rationale and environment may distort causal relations between substructures and labels, causing models to learn spurious correlations. Therefore, ensuring latent space regularity, chemical interpretability, and task-aware constraints is essential for the safe and reliable use of latent space data augmentation.

#### 4.2.3 Data Augmentation and Data Generation in Structure Space

To create molecules with new structures, molecular data augmentation can proceed through either substructure substitution or through generative modeling. Substructure-based approaches modify existing molecular graphs by altering local motifs while maintaining overall connectivity. Examples in traditional medicinal chemistry include the well-known approaches of replacing carbons or hydrogens with different heteroatoms or functional groups by isosters. In the area of generative data augmentation, GraphCrop^297^ extracts contiguous subgraphs centered on specific atoms to simulate structural cropping, whereas M-Evolve^298^ perturbs edges within identified motifs through stochastic sampling. MoCL^299^ integrates chemical knowledge by replacing functional groups with chemically similar analogues or bioisosteres, and GRIN^300^ exploits the repetitive monomer patterns in polymers to build invariant representations through motif repetition. While these methods efficiently expand molecular diversity, they rely on the assumption that substituting substructures does not alter the molecular label, an assumption that may not hold, since the contribution of functional groups can vary across different chemical properties and prediction tasks. Another limitation is that substructure-based substitution focuses on local changes and cannot capture global patterns in the data augmentation.

In contrast, generative models construct complete and valid molecular structures directly from learned data distributions, offering greater flexibility beyond that of local modifications. Depending on the molecular representation, generative methods can be string-based (e.g., SMILES and SELFIES)^83^ or graph-based,^290^ as shown in Table 2. Early models such as PI1M^289^ used LSTM architectures to expand polymer monomer diversity, while MolGAN^290^ introduced adversarial learning with property-guided reinforcement to generate molecules that satisfy target objectives. JT-VAE^106^ combined graph and tree representations, enabling valid molecule generation through latent-variable control. More recently, diffusion models have emerged as powerful frameworks for modeling complex molecular distributions. GDSS^291^ adapts continuous diffusion with Gaussian noise to molecular graphs via score-based dynamics, and DiGress^292^ extends this idea to discrete atoms and bonds by defining a transition matrix over the state frequencies. These models can generate large numbers of unlabeled molecules to augment training data, but conditional generation techniques^301,302^ are required to control the generation process in data augmentation scenarios, such as in-class augmentation or the generation of additional data for low-resource regions.^303^

**Table 2:** Representative Generative Models for Molecular Data Augmentation

Model	Architecture	Molecular Representation
LSTM^289^	Recurrent Neural Network	String-based
MolGAN^290^	Generative Adversarial Network	Graph-based
JT-VAE^106^	Variational Autoencoder	Graph and tree-based
GDSS^291^	Diffusion Model	Graph-based
DiGress^292^	Diffusion Model	Graph-based
Graph DiT^293^	Diffusion Model	Graph-based
MolT5 ^294^	Transformer	String-based
LlaSMol^295^	Transformer	String-based
LlaMole^296^	Transformer	Graph-based

For conditional molecular generation, early work focused on molecular optimization, which transforms the desired property into a normalized score (typically scaled between 0 and 1) and then iteratively optimizes the molecular structure toward a target score of 1 using an oracle function that evaluates the molecule at each step. Various molecular representations, such as SMILES strings^304^ and molecular graphs,^305^ have been explored in combination with generative models like VAEs^106^ and sampling-based methods.^306^ Subgraphs associated with specific properties have also been used as rationales to guide the optimization process.^106^ Optimization algorithms used in this setting include genetic algorithms,^305^ Bayesian optimization,^307^ and hill climbing, a variant of REINFORCE.^308^ Mollaysa et al.^309^ applied reinforcement learning to maximize the expected reward in sequence-based conditional molecular generation. LaMOO^310^ explored a 32-dimensional latent space to generate molecules with targeted properties, whereas DST^311^ used gradient-based optimization over discrete molecular graphs with scaffold trees. Zhu et al.^312^ proposed a multiobjective Bayesian optimization approach using hypernetwork-based GFlowNets. However, in multiobjective optimization, determining the appropriate trade-off between multiple target properties remains challenging and is still underexplored. Most existing optimization methods rely on a large number of oracle function calls (often exceeding 10 000 ^313^), which may be infeasible when such oracles are inaccessible or expensive during training.

Another line of molecular conditional generation focuses on jointly modeling the label–structure distribution using generative models such as LSTMs or diffusion models.^293,314^ Unlike prediction models, where molecular structures are used to predict labels, these generative models take the desired property labels as input and generate molecules that satisfy those conditions. A key challenge in this setting is generating molecules under multiple objective constraints.^315^ To address this, Graph Diffusion Transformers (Graph DiTs)^293^ learn representations of the input conditions and extend the diffusion transformer architecture to perform denoising on discrete molecular structures. They have demonstrated strong performance under multiproperty constraints. Graph DiTs use condition representations to guide the diffusion process. An alternative strategy is to use an auxiliary prediction model to provide guidance during diffusion. In image generation, classifier-guided diffusion^302^ uses a classifier that takes a noisy image and the corresponding noise level (or diffusion time step) as input, predicts the class label, and computes input gradients to refine the denoised structure. DCT^278^ extends this idea to molecules by incorporating prediction-guided diffusion into a data augmentation framework. It uses a pretrained diffusion model to modify input molecule–label pairs in downstream prediction tasks, generating task-specific molecules to improve prediction performance.

Beyond generative modeling of molecules, large language models (LLMs) have emerged as useful foundation models across many domains, including molecular understanding through string-based representations such as SMILES and SELFIES.^294–296,316^ LLMs can also be instructed through natural language to generate new molecules, providing a promising approach to augment training data sets and mitigate data sparsity.^294,296^ However, current LLMs struggle to interpret complex molecular instructions when fine-tuned on limited data sets.^274,296^ Many existing molecular instruction data sets lack informativeness, e.g. molecular descriptions often focus on the source or origin of a compound rather than detailing its structural features or linking them to target properties as presented in PubChem.^316,317^ Additionally, these data sets often lack diversity, covering narrow chemical domains (e.g., small-molecule drug-like molecules) with limited generalizability to broader applications such as polymeric materials. Moreover, it remains challenging to control language models to generate or edit molecular structures that satisfy specific objectives at a fine-grained level.^296,318^

Chemical validity does not mean that a molecule is realistic for synthesis. Another important dimension of molecular realism is the synthesizability. Several lines of research have explored synthesizable molecular generation using either bottom-up^319,320^ or top-down approaches. Bottom-up methods start from purchasable building blocks^320^ and construct synthetic pathways by applying consecutive chemical reactions to generate the final product. While this ensures that the designed molecule is synthesizable, the search space is constrained by known reactions and available starting materials, limiting the flexibility in molecular design. In contrast, top-down methods^296,321^ follow a retrosynthetic planning paradigm: they first design the target molecular structure and then search for a synthesis pathway by recursively decomposing the molecule into smaller available precursors through known reactions. However, such methods do not guarantee that a valid synthesis route exists for a designed structure. To address this, some approaches aim to find analogues or alternatives to the designed molecules that are more readily synthesizable.^321^ In the context of data augmentation, incorporating synthesizability as a regularization constraint can improve the quality of augmented molecules. By encouraging the generation of chemically realistic and synthesizable structures, this constraint helps to produce more useful and reliable training data.

##### 4.2.3.1 Chemistry-Specific Evaluation Metrics for Synthetic Data Quality

Existing molecular generation benchmarks such as GuacaMol and MOSES provide many metrics, including validity, novelty, uniqueness, internal diversity, and Fréchet ChemNet Distance^304^ that can quantify whether a model produces chemically parseable structures and whether a generated set resembles a reference distribution.^322,323^ However, high benchmark scores do not guarantee that the new synthetic molecules have trustworthy labels or that they are chemically realistic under the constraints of synthesis and mechanism.^324^ For chemistry tasks, evaluation should go beyond generic diversity indices to include: (i) *validity* checks that match the representation and task, including stereochemical consistency where relevant (not only valence checks); (ii) *property fidelity*, measured by agreement of conditional target distributions and key physchem covariates, and, when possible, spot checking synthetic labels with a higher fidelity oracle;^293,314,325^ (iii) *mechanistic and synthetic plausibility*, using synthesizability proxies such as SAScore, and stronger learned or route linked scores such as SCScore and RAscore where appropriate;^326–329^ and (iv) *downstream task utility*, quantified by improvements on held out real test sets (and specifically in the rare target ranges driving augmentation), rather than by distribution similarity alone.^278^ Together, these criteria motivate chemistry specific synthetic data metrics that are grounded in label credibility, feasibility, and predictive value.

##### 4.2.3.2 When is Data Augmentation Sufficient, and When Is Generative Modeling Necessary?

Data augmentation is sufficient when transformations are close to label-invariant and remain within data distribution so that augmented samples are alternative views of known chemistry rather than new, weakly labeled structures. For example, randomized SMILES expands supervision without changing molecular identity and has shown measurable gains in low-data prediction settings. The task-specific molecular augmentation libraries improve generalization by controlled perturbations.^277,330^ In contrast, generative modeling becomes necessary when the project requires molecules in sparse or unseen regions of property space where local perturbations cannot supply informative coverage or when the desired targets demand new scaffolds and global structural changes that are not reachable by fragment substitution or small feature masking without changing the label in an uncontrolled way. In these regimes, conditional generation and semisupervised synthesis methods can add new molecules by constructing structures or graphs targeted to underrepresented labels. They also increase the need for stronger evaluation, since standard novelty and diversity metrics alone do not protect against unrealistic chemistry, shortcuts, or label mismatch.^267,324^ A practical framing is to treat augmentation as a first choice for robustness and small data regularization under clear invariances and to reserve generative modeling for cases of structural coverage gaps, while tightening quality control toward property fidelity, feasibility, and measured downstream gains in the rare value regions that motivated data generation.

##### 4.2.3.3 Risks and Future Direction

Generative modeling in structure space introduces risks related to chemical validity, synthesizability, and controllability. Although models such as diffusion networks and large language models can generate chemically valid SMILES or graphs, many produced molecules are unstable, unsynthesizable, or not suitable for the desired target properties. Property- or reward-guided generation may overfit target objectives, producing structures that maximize predicted scores but violate physical or chemical constraints. In multiobjective or text-conditioned generation, inconsistent property trade-offs or ambiguous language inputs can result in hallucinated or chemically invalid molecules. Future work should incorporate constraints reflecting synthetic accessibility, stability, and chemical feasibility directly into generative objectives and integrate feedback from property predictors, reaction models, and retrosynthesis planners to ensure realistic, interpretable, and reliable molecular generation in data augmentation.

##### 4.2.3.4 Chemistry Pushes AI Boundaries

Chemistry’s long-tailed property distributions and the unique challenges of continuous-outcome imbalance have driven methodological innovation in both supervised learning and generative modeling. Unlike classification tasks where class imbalance can be addressed through reweighting or oversampling discrete labels, chemical property prediction involves continuous targets (yields, binding affinities, reaction barriers) where underrepresented but scientifically critical regions (high yields, extreme selectivities, rare property combinations) carry disproportionate value. The mismatch between data prevalence and scientific importance necessitated the development of imbalanced regression techniques such as relevance-weighted losses, local resampling in continuous spaces, and domain-specific augmentation strategies that extend beyond standard regression frameworks. Similarly, the requirement to generate chemically valid, diverse, and property-targeted molecules under sparse supervision with accurately predicted properties has spurred advances in structure-space generation (graph diffusion models, fragment-based sampling, retrosynthesis-aware generators) and physics-informed augmentation (conformer sampling, reaction-mechanism-guided perturbations). These methods, which balance fidelity to chemical constraints with exploration of underrepresented regions, offer generalizable strategies for data generation and rebalancing in other scientific domains characterized by continuous outputs, physical constraints, and value-imbalanced distributions. Finally, it should be noted that data augmentation strategies rely on continuous relationships of structure and properties. This is not always the case in chemistry as demonstrated e.g. by nonobvious activity cliffs.

## 5 Reaction Prediction

Reaction prediction encompasses two closely related tasks that are at the core of synthetic chemistry: *retrosynthesis*, which infers viable precursors to synthesize a desired target molecule using known reaction precedent, and *forward reaction prediction*, which forecasts the product(s) formed by a given set of reactants, reagents, catalysts, and conditions, as seen in Figure 9. These dual tasks mirror the cognitive strategies of organic chemists: reasoning either from cause to effect or from goal to precursors. Historically, these problems were addressed through expert-curated rules and mechanistic heuristics, but the proliferation of reaction databases and machine learning has shifted the field toward data-driven and neural networks. Modern reaction prediction systems leverage symbolic logic, graph representations, and large language models to capture chemical reactivity, evaluate synthetic feasibility, and propose practical multistep synthetic routes. In this section, we examine retrosynthesis and forward synthesis both conceptually and computationally, highlighting how they inform one another and evolve in computer-assisted synthesis planning (CASP).

**Figure 9:**
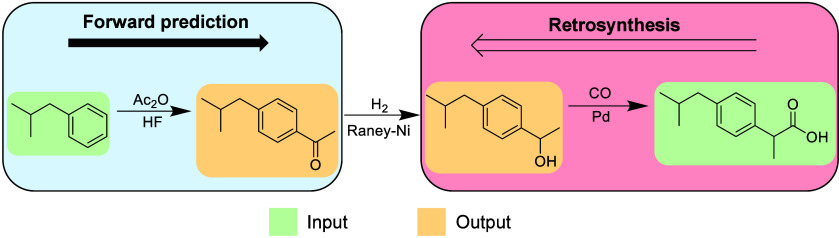
Overview of the reaction prediction framework for synthesizing ibuprofen with the Boots–Hoechst–Celanese (BHC) process, including both retrosynthesis starting from ibuprofen to isobutylbenzene and forward reaction prediction reversely.

### 5.1 Retrosynthesis

#### 5.1.1 Pre-ML Expert Systems

Before the era of data-driven or machine learning approaches, computer-assisted retrosynthesis was guided by *expert systems*—programs that tried to imitate the reasoning of skilled organic chemists. These systems relied on large collections of reaction rules and chemical heuristics, rather than statistical models, to plan synthetic routes.

As shown in Figure 10, the earliest example was the OCSS (Organic Chemical Simulation of Synthesis) system developed by Corey and Wipke in 1969,^331^ which introduced the idea of representing chemical reactions as “transforms.” By working backward from a target molecule, OCSS could suggest plausible disconnections based on encoded reaction patterns. This concept matured into LHASA (Logic and Heuristics Applied to Synthetic Analysis),^332^ which became the most influential pre-ML retrosynthesis system. LHASA combined logical rules with heuristic “expert knowledge,” allowing chemists to interactively explore alternative synthetic routes and refine them using domain insight.

**Figure 10:**
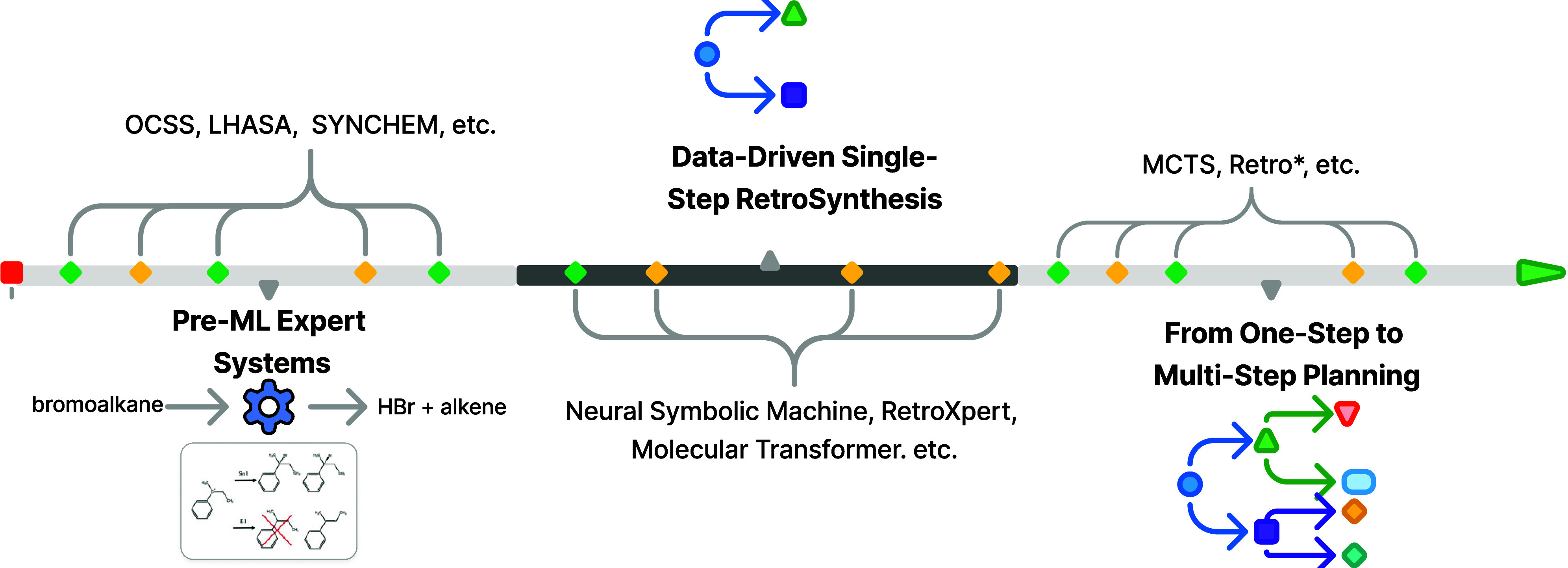
Overview of the evolution of retrosynthesis systems from premachine learning expert systems (e.g., OCSS,^331^ LHASA,^332^ SYNCHEM^333,334^ ) to modern data-driven and neural network approaches. The figure illustrates the transition from rule-based and template-driven one-step prediction toward neural-symbolic, transformer-based, and multistep planning models such as Neural Symbolic Machine,^335^ RetroXpert,^336^ Molecular Transformer,^163^ and Monte Carlo Tree Search (MCTS) variants like Retro*.^337^ Example reaction: the addition of HBr to an alkene forming a bromoalkane.

Other research groups have extended these ideas. SECS (Simulation and Evaluation of Chemical Synthesis)^338^ formalized reaction transforms as production rules and could simulate both forward and backward syntheses. The SYNCHEM and SYNCHEM-2 systems^333,334^ emphasized heuristic search and evaluation of synthetic trees to identify feasible synthetic plans. SYNGEN^339^ introduced a systematic method for generating synthesis trees, focusing on the logic of multistep design.

These expert systems collectively defined the foundation of computer-aided synthesis design, see Table 3 for a summary. They captured chemical reactivity and synthetic logic in symbolic form, demonstrating that much of the retrosynthetic reasoning could be formalized. However, their dependence on hand-coded rules and limited adaptability to new chemistry ultimately constrained their scope, motivating the emergence of machine-learning methods that could learn chemical reactivity directly from data.

**Table 3:** Representative Pre-ML Rule-Based Expert Systems for Retrosynthesis

System	Paradigm	Scope
OCSS^331^	Rule-based expert system	Multistep
LHASA^332^	Rule-based expert system	Multistep
SECS^338^	Rule-based expert system	Multi/bidirectional
SYNCHEM(−2)^333,334^	Rule-based expert system	Multistep
SYNGEN^339^	Rule-based expert system	Multistep

#### 5.1.2 Data-Driven Single-Step Retrosynthesis

With the growth of large reaction databases such as USPTO, Reaxys, and Pistachio, retrosynthetic analysis began to shift from rule-based reasoning to data-driven learning. Rather than relying on expert-coded transforms, models learned reactivity patterns directly from thousands of recorded reactions, enabling automated prediction of precursors for a given product. This transition marks the beginning of modern *single-step retrosynthesis*—predicting reactants for one target molecule, one step at a time, based on empirical reaction data.

Early approaches were framed as a pattern recognition task. Neural networks or ensemble models were trained to classify the most likely reaction template from a set of known transformations, as seen in the works.^340,341^ These template-based methods combined learned pattern recognition with existing reaction rules, offering both chemical interpretability and improved scalability compared to purely heuristic systems. However, they were constrained by the completeness of the underlying data sets, which require multiple examples to generate high-quality templates, and the resulting low granularity of their template libraries.

Subsequent studies explored *template-free* paradigms, inspired by advances in neural machine translation. By representing molecules as SMILES strings, these works^342,343^ trained sequence-to-sequence models that directly translated a product into plausible reactants, bypassing the need for explicit templates. Transformer-based architectures such as RetroXpert and Molecular Transformer^163,336^ further improved performance by capturing long-range dependencies and reaction context. These models treat retrosynthesis as a language translation problem—translating the “product language” into the “reactant language”—and learn chemical grammar implicitly from data.

While data-driven methods achieved substantial gains in accuracy and generalization, they also introduced new challenges. Models often suffer from limited chemical interpretability, sensitivity to data set biases, and difficulties in enforcing chemical validity or reaction feasibility. To address these issues, hybrid methods combining learned reaction priors with symbolic verification or graph-based constraints have emerged.^344,345^ Nevertheless, data-driven single-step retrosynthesis remains a cornerstone of modern computer-aided synthesis, forming the foundation for multistep planning systems and reinforcement-learning-based route optimization.

#### 5.1.3 From One-Step to Multi-Step Planning

While single-step retrosynthesis models can predict the immediate precursors of a target molecule, true CASP requires assembling these local predictions into feasible multistep routes. The transition from one-step prediction to multistep *planning* introduces new challenges in search, optimization, and chemical consistency. Each predicted intermediate can itself become a new target, creating a branching search tree whose depth and breadth grow combinatorially with molecular complexity, quickly reaching a point at which it becomes computationally intractable.

Early data-driven planners combined single-step models with heuristic search algorithms such as Monte Carlo tree search (MCTS) or best-first search, using reaction likelihoods or learned scores as guidance. For example, Segler et al.^335^ demonstrated that coupling a neural template predictor with MCTS could efficiently explore large reaction networks, pruning implausible routes, while maintaining chemical diversity. Such a hybrid system integrated learned one-step models with symbolic reasoning, marking a practical bridge between AI planning and chemical synthesis.

More recent frameworks formulate multistep retrosynthesis as a sequential decision-making problem. A reinforcement learning (RL)-based planner, such as Retro* and retrosynthesis through simulated experiments,^337,346^ learns to optimize global objectives—such as route length, synthetic accessibility, or commercial availability of starting materials—rather than local prediction accuracy alone. These systems maintain a search graph over possible intermediates and employ policy networks or value functions to prioritize promising pathways. Other approaches introduce retrosynthetic grammars or cost-aware graph search,^347–350^ explicitly accounting for reaction feasibility, reagent price, and yield data.

Despite notable advances, multistep planning remains a frontier problem. Current systems are limited by error propagation from imperfect one-step models, incomplete coverage of the reaction space, and the difficulty of integrating kinetic or thermodynamic feasibility into search heuristics. Nonetheless, the move from isolated one-step predictions to holistic route planning has redefined computer-aided synthesis design—from static reaction forecasting toward dynamic, goal-oriented reasoning capable of producing experimentally executable synthetic plans, see Table 4 for a summary of single-step retrosynthesis and multistep planning systems.

**Table 4:** Representative Data-Driven Single-Step Retrosynthesis Models and Multi-Step Planning Systems

Model/Framework	Type	Scope
Template ranking (Coley17)^340^	Template-based	Single-step
Neural rule scoring^341^	Template-based	Single-step
Seq2Seq (Nam and Kim)^342^	Template-free (RNN)	Single-step
Seq2Seq (Liu17)^343^	Template-free (RNN)	Single-step
RetroXpert^336^	Semitemplate/Editing	Single-step
Molecular Transformer^163^	Transformer	Single-step
Graph-to-Graphs (G2Gs)^344^	Graph editing	Single-step
RetroTRAE/Substructure^345^	Fragment-level	Single-step
Neural + MCTS Planner^335^	Hybrid search	Multistep
Retro*^337^	Learned A*/AND-OR	Multistep
RL-based planning^346^	RL planner	Multistep
AiZynthFinder^351^	Toolkit	Multistep
ASKCOS^352^	Platform	Single-step and multistep
Syntheseus^353^	Benchmarking framework	Evaluation only
RetroComposer^354^	Template generation	Single-step

#### 5.1.4 Emerging Directions

Recent retrosynthesis research has shifted focus from maximizing top-*k* exact-match accuracy toward more holistic indicators of synthetic quality. Evaluation objectives now emphasize round-trip validation, route diversity, and step- or cost-aware metrics that correlate more strongly with real-world feasibility. Frameworks such as AiZynthFinder,^351^ Retro*,^337^ and Syntheseus^353^ have begun to incorporate multiobjective criteria—yield, reagent cost, route length, or reaction probability—linking algorithmic performance to practical synthesis outcomes.

Parallel advances in editing-based retrosynthesis^355–359^ and data-driven template generation have broadened chemical coverage beyond static rule libraries.^354,360–362^ By learning generalized reaction transformations directly from atom-mapped data, these methods enable flexible exploration of novel chemical space while maintaining chemical validity through explicit valence and reactivity constraints. This evolution blurs the line between reaction prediction and retrosynthesis, allowing models to reason over transformations rather than memorized templates.

At the system level, unified platforms have emerged that integrate multiple one-step models, ranking functions, and search strategies within a single, reproducible framework. Such a modular design encourages controlled ablation, fair benchmarking, and systematic evaluation across diverse chemical domains. Open initiatives such as ASKCOS^352^ and AiZynthFinder^351^ demonstrate a growing commitment to transparency and standardization, while new benchmarks emphasize data provenance, realistic chemical constraints, and route-level reproducibility.

Another emerging frontier is the integration of large language models (LLMs) and multiagent reasoning frameworks into synthesis planning.^131,363,364^ These systems can propose, critique, and refine disconnections in natural language, bridging symbolic and neural reasoning. Yet, claims of “chemical reasoning” must be interpreted carefully: standardized, route-level benchmarks and counterfactual tests are needed to disentangle true mechanistic understanding from data set memorization.

For computer scientists, retrosynthesis now presents a rich interdisciplinary testbed for search–learning integration, causal inference, and human–AI collaboration. Future progress will depend on models that combine data-driven pattern discovery with symbolic constraint solving, algorithms that optimize across multiple synthetic objectives simultaneously, and interfaces that support iterative dialogue between chemists and AI planners. The overarching challenge and opportunity are to transform retrosynthesis from a predictive modeling task into an interactive reasoning process that unifies algorithmic efficiency, chemical validity, and human interpretability.

### 5.2 Reaction Forward Prediction

Reaction product prediction lies at a fundamental part of chemistry, which aims to predict the outcome of a set of given chemicals, including reactants and reagents, as shown in Figure 11. The ability to accurately predict chemical reactions not only accelerates the development of new compounds but also reduces the reliance on costly and time-consuming in-lab procedures, which is thus beneficial in an acceleration for drug discovery and synthetic path planning.^331,365,366^

**Figure 11:**
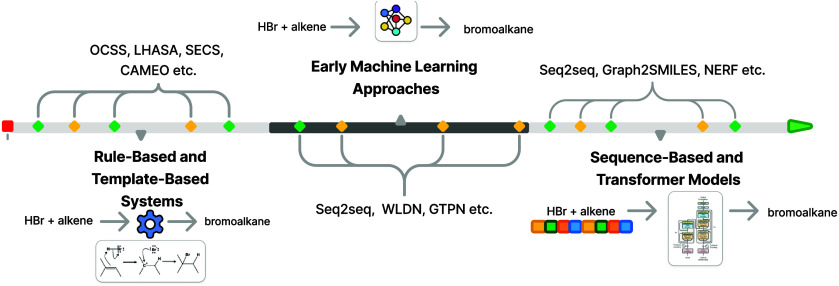
Progression of reaction product prediction paradigms from rule-based and template-based systems (e.g., OCSS,^331^ LHASA,^332^ SECS,^338^ CAMEO^367^) to early machine learning and modern sequence-based models. The figure highlights the shift from hand-crafted reaction templates to data-driven architectures such as Seq2Seq,^342^ WLDN,^368^ Graph2SMILES,^369^ and NERF.^370^ The example reaction illustrates the transformation of an alkene into a bromoalkane via HBr addition.

#### 5.2.1 Rule-Based and Template-Based Systems

Before data-driven learning became dominant, reaction forward prediction was chiefly approached with knowledge-based systems that encoded mechanistic heuristics and reaction templates curated by experts. Representative systems include CAMEO,^367^ EROS,^371^ ROBIA,^372^ the fully theoretical BEPPE program,^373^ and graph-rewriting “toy chemistry” models.^374^ These systems captured organic chemistry know-how, formalized in functional-group perception, reactive-site ranking, electron-pushing patterns, and pericyclic rules, and combined them with filtering or ranking via physicochemical or energetic criteria. They offered mechanistic interpretability, transparent rationales, and fast inference once rules were in place. However, coverage and maintenance needed manual curation and were thus bottlenecks: expanding to new reaction classes or rare reagents required writing and validating new rules/templates and ensuring compatibility with existing rules. In addition, extrapolation beyond the encoded library was unreliable. As a result, scalability and generalization lagged compared to later, data-driven methods.

Mechanistic breadth varied across systems. CAMEO emphasized mechanistic selection among competing pathways and provided product rankings with explanatory comments and estimated thermochemistry (spanning nucleophilic/electrophilic processes, pericyclic reactions, radicals, and carbene chemistry). EROS combined multistep, graph-based rule libraries with auxiliary modules (heats of formation, physicochemical properties, and kinetic simulations) to constrain and prioritize outcomes, while also informing synthesis design. ROBIA integrated a rule-driven enumerator with conformational searches and *ab initio* single-point energies to filter intermediates and rank plausible products under specified conditions. BEPPE explicitly pursued a “theoretical” route: it generated candidate products without fitting to experimental outcomes and selected among them using computable reactivity descriptors, with early demonstrations on Diels–Alder chemistry. A parallel line encoded electron flow and “arrow pushing” directly, for example, implemented over 1500 transformation rules to predict mechanisms and products.^375^

In summary, pre-ML rule/template systems delivered mechanistic transparency and chemically sensible reasoning traces, but their dependence on handcrafted knowledge limited scalability, domain transfer, and robustness to untemplated chemistry—limitations that motivated the subsequent shift toward statistical and neural methods.

#### 5.2.2 Early Machine Learning Approaches

The emergence of large, text-mined reaction corpora (notably the USPTO extractions by Lowe^376^) enabled data-driven models to move beyond handwritten rules and fixed libraries of templates. Early pipelines coupled learned pattern recognition with either explicit templates or limited, enumerative chemistry:^377^ trained a neural classifier on concatenated (reactant, reagent) fingerprints to predict reaction type, then invoked a matched SMARTS template to generate products, establishing the “learn-to-choose, rule-to-apply” paradigm^342^ instead reframed forward prediction as sequence-to-sequence translation from “reactants+reagents” SMILES to product SMILES, dispensing with expert templates altogether. These works demonstrated that modest neural models can generalize beyond curated rule bases when supported by sufficiently large reaction text corpora.

Template-augmented learning matured with the work of Green, Jensen and co-workers,^366^ who automatically mined reaction SMARTS from atom-mapped USPTO data and trained a neural ranker over self-generated candidate products, substantially improving top-*k* accuracy while retaining mechanistic interpretability at the level of reaction cores. In parallel, knowledge-graph formulations^378^ treated product prediction as link prediction over a global molecule–reaction graph (millions of nodes/edges), yielding competitive accuracy, extrapolation to postcutoff reactions, and a transparent reasoning substrate.

Moving beyond templates, graph neural networks modeled reactions as sparse edits on molecular graphs. Jin et al.^368^ introduced the Weisfeiler–Lehman Network (WLN) to localize reaction centers and the WLDN to rerank enumerated candidates, achieving sizable gains and speedups over template methods. Follow-ups explored mechanism-level supervision and decision-process formalisms: Fooshee et al.^379^ trained deep models to predict elementary arrow-pushing steps and chain them into global outcomes, while the Graph Transformation Policy Network (GTPN)^380^ learned sequences (sets) of bond changes with reinforcement learning, avoiding a fixed number or ordering of edits.

#### 5.2.3 Sequence-Based and Transformer Models

The introduction of sequence-based molecular representations, such as SMILES, enabled neural models to treat reaction prediction as a translation problem. Early sequence-to-sequence (seq2seq) frameworks based on recurrent neural networks (RNNs) learned to map concatenated reactant and reagent strings to product SMILES, marking the first generation of data-driven reaction translators.^342,381^ With the development of self-attention architectures, transformer-based models further enhanced this direction, capturing long-range dependencies and complex rearrangements within chemical strings.^163,382,383,386^ These autoregressive models generate product tokens sequentially and achieve strong top-*k* accuracy on large-scale reaction data sets such as USPTO,^368,387^ establishing a solid foundation for reaction language modeling.

As attention has shifted toward structure-preserving representations, graph-based formulations have become increasingly prominent. In Graph2SMILES^369^ architectures, reactant molecules are encoded as graphs through message-passing neural networks, while product SMILES are decoded through transformer-like generators, combining graph structural priors with the expressiveness of language models. Building upon this hybrid design, fully Graph2Graph models represented both reactants and products as molecular graphs, treating chemical transformations as a set of learned graph edits.^380,384^ Representative approaches such as the Graph Transformation Policy Network (GTPN) and MEGAN model reactions as sequences of bond changes, each predicted from atom-level contexts and reaction states. This paradigm provides an explicit, mechanistically aligned view of chemical reactivity.

Subsequent developments extended these ideas toward nonautoregressive formulations. Instead of predicting one bond change at a time, models such as NERF^370^ and ReactionSink^385^ infer all reaction edits in a single forward pass, framing chemical transformations as global electron redistributions. These one-shot models integrate physically inspired constraints such as electron counting and symmetry, improving both efficiency and interpretability. Empirical results demonstrate that such nonautoregressive graph frameworks reach accuracy comparable to Transformer-based SMILES models while substantially reducing inference time, achieving more than an order-of-magnitude speedup.^370^ See Table 5 for a summary of neural architectures for reaction forward prediction.

**Table 5:** Representative Neural Architectures for Reaction Outcome Prediction (2016–2025)^a^

Model	Input Modality	Output Modality	Model Type	Template-Free?
Neural Reaction Fingerprint^377^	Fingerprint	SMILES	MLP	√
NMT-Reaction^342^	SMILES	SMILES	Seq2Seq (RNN)	√
Reaction Predictor^366^	SMILES	SMILES	MLP	√
Neural-Symbolic Reaction Predictor^378^	Graph	Graph	GNN	√
WLDN^368^	Graph	Graph	GNN	√
Deep Reaction Predictor^379^	Graph + SMILES	Graph/SMILES (step-level)	Hybrid (MLP + LSTM)	√
GLN^380^	Graph	Graph	GNN + RL	√
Molecular Transformer^381^	SMILES	SMILES	Seq2Seq (LSTM)	√
Molecular Transformer-UQ^163^	SMILES	SMILES	Transformer	√
Augmented Molecular Transformer^382^	SMILES (augmented)	SMILES	Transformer	√
Chemformer^383^	SMILES	SMILES	Transformer (BART)	√
MEGAN^384^	Graph	Graph	GNN (GAT)	√
NEC-GAE^370^	Graph	Graph	GNN + Transformer + VAE	√
DSMG^385^	Graph	Graph	GNN + Transformer + VAE (Sinkhorn)	√

a Models are grouped by input/output modality and major neural component type.

#### 5.2.4 Trade-Offs between Template-Based and Template-Free Methods

Template-based reaction prediction provides explicit and chemically interpretable graph transformations, which improve reliability, controllability, and auditability, particularly in low-data regimes or narrowly scoped reaction families. Because templates encode expert-derived bond edit rules, they impose strong chemical validity constraints and enable easier debugging and interpretation. However, template-based approaches are inherently limited by template coverage and require continuous extraction, deduplication, and maintenance as new reactions emerge. In contrast, template-free methods eliminate coverage bottlenecks and can generalize beyond predefined transformation rules by learning continuous representations of reactivity patterns. This makes them more suitable for large-scale, diverse data sets and open-ended prediction settings. The trade-off is that template-free models typically require more training data and careful optimization and may lack explicit mechanistic interpretability unless additional constraints or reaction center supervision are incorporated.

#### 5.2.5 Trade-Offs between Graph-Based and Sequence-Based (SMILES) Representations

Graph-based reaction prediction models explicitly operate on molecular topology, enabling the direct modeling of atom-level interactions, bond connectivity, and local structural environments. This representation is particularly advantageous when prediction depends on precise reaction center identification, stereochemical consistency, or fine-grained structural constraints. Graph-based models are also inherently invariant to SMILES linearization choices, improving robustness to representation artifacts. In contrast, sequence-based methods using SMILES representations benefit from the scalability and optimization advantages of transformer architectures as well as the availability of large-scale pretraining strategies. When sufficient data are available, SMILES-based sequence models can effectively learn reaction prediction as a translation task and achieve strong empirical performance. However, they may be sensitive to tokenization and canonicalization choices and do not explicitly encode molecular topology, unless additional alignment or structural supervision is introduced.

#### 5.2.6 Summary of Practical Considerations

Overall, template-based methods offer higher interpretability and reliability in constrained domains but suffer from limited coverage and scalability, whereas template-free methods provide greater flexibility and generalization at the cost of higher data and training requirements. Similarly, graph-based models are preferable when structural fidelity and reaction center accuracy are critical, whereas sequence-based models are advantageous when leveraging large-scale pretraining and scalable sequence modeling infrastructure. Recent advances increasingly combine these paradigms, incorporating structural inductive biases into transformer architectures to balance chemical fidelity, generalization, and scalability.

#### 5.2.7 Emerging Directions

Recent advances in reaction forward prediction underscore a broader shift from local, pairwise modeling of chemical transformations to representations that capture collective, context-dependent reactivity on multiple structural and mechanistic scales. Three converging directions illustrate this transition.

##### 5.2.7.1 Multi-Scale and Mechanistically Grounded Representations

While most current neural models operate on molecular graphs defined by pairwise bonds, many organic reactions involve higher-order structural motifs, electron flow involving multiple atoms and electron pairs, and multicenter rearrangements that are challenging to represent with simple edge-level connectivity. Emerging graph formalisms such as *hypergraphs*, *simplicial complexes*, and *higher-order message passing networks* ^388–392^ allow direct encoding of reaction centers, delocalized electron systems, and functional-group “modules” as intrinsic units of reasoning rather than as emergent patterns from pairwise interactions. These high-order network representations provide an approach to integrate mechanistic domain knowledge into data-driven models, enabling improved robustness across substitution patterns, conformational regimes, and underexplored reaction families.

##### 5.2.7.2 Condition- and Environment-Aware Prediction

Forward prediction models increasingly incorporate explicit reaction conditions such as solvent, catalyst, temperature, concentration, and stereochemical environment.^393–396^ Large-scale extractions of condition-annotated reactions and structured lab-procedure corpora (e.g., curated ELNs and patent text mining) are enabling models that treat conditions not as auxiliary tokens but as thermodynamic and kinetic parameters influencing the reaction energy landscape. Coupling these condition-aware encodings with high-order representations offers a pathway toward models that can not only reproduce observed products but also *predict outcomes under modified conditions* and guide optimization of chemo-, regio-, and stereoselectivity.

##### 5.2.7.3 Unifying Forward Prediction, Retrosynthesis, and Reaction Planning

Forward prediction historically developed in parallel with retrosynthesis and multistep route design. However, recent work suggests these tasks can be treated as dual inference problems over a shared reaction network.^397^ In this view, forward prediction becomes an integral component of *bidirectional* synthesis reasoning, supporting dynamic search strategies akin to Monte Carlo planning or adaptive testing, where local transformation scores inform global path optimality.^352,398–400^

### 5.3 Chemistry Pushes AI Boundaries

The “combinatorial explosion” in complexity of reaction prediction and retrosynthesis has necessitated fundamental innovations in neural architecture and search algorithms that extend well beyond chemistry. Unlike traditional supervised learning tasks involving single molecules, chemical reactions inherently involve multibody interactions among multiple reactants, reagents, catalysts, and solvents, whose collective behavior cannot be decomposed into pairwise relationships.^388^ This irreducible many-body structure drove the development of hypergraph neural networks, reaction-center attention mechanisms, and higher-order message-passing schemes capable of representing joint interactions among arbitrary subsets of molecular participants. Similarly, the exponential search space of multistep retrosynthesis, where each intermediate spawns branching disconnections and chemically invalid paths must be pruned efficiently, motivated the integration of Monte Carlo tree search with learned neural guidance, best-first planning with value-function approximation, and reinforcement learning frameworks that optimize global route objectives rather than local one-step accuracy. The requirement to balance exploration (testing novel disconnections) with exploitation (refining promising pathways) under expensive oracle queries (experimental validation, DFT calculations) has made reaction planning a canonical testbed for neural-guided search, active learning, and constrained optimization. These strategies, refined in the context of synthesis planning, now inform AI systems across domains requiring structured combinatorial search under uncertainty.

## 6 Chemical Reasoning: From Induction to Deduction

As AI and ML methods have matured, the problem space now available to computational chemists has broadened. No longer limited to well-conditioned x to y mappings replete with data (i.e., inductive problems), scientists now advance solutions to ill-defined (deductive) questions: problems which have no clean analytic solution, are under-determined, contain contradictory information, or whose underlying mapping is nonfunctional^401,402^ (Figure 12). Deductive problems require solutions that operate by dynamically integrating diverse data streams, such as analytical spectra, reaction conditions, or kinetic data, not merely as extra features, but as logical constraints. Increasing success in these problems advances beyond statistical correlation toward more complex emulation of an expert chemist’s use of evidence.^401–403^

**Figure 12:**
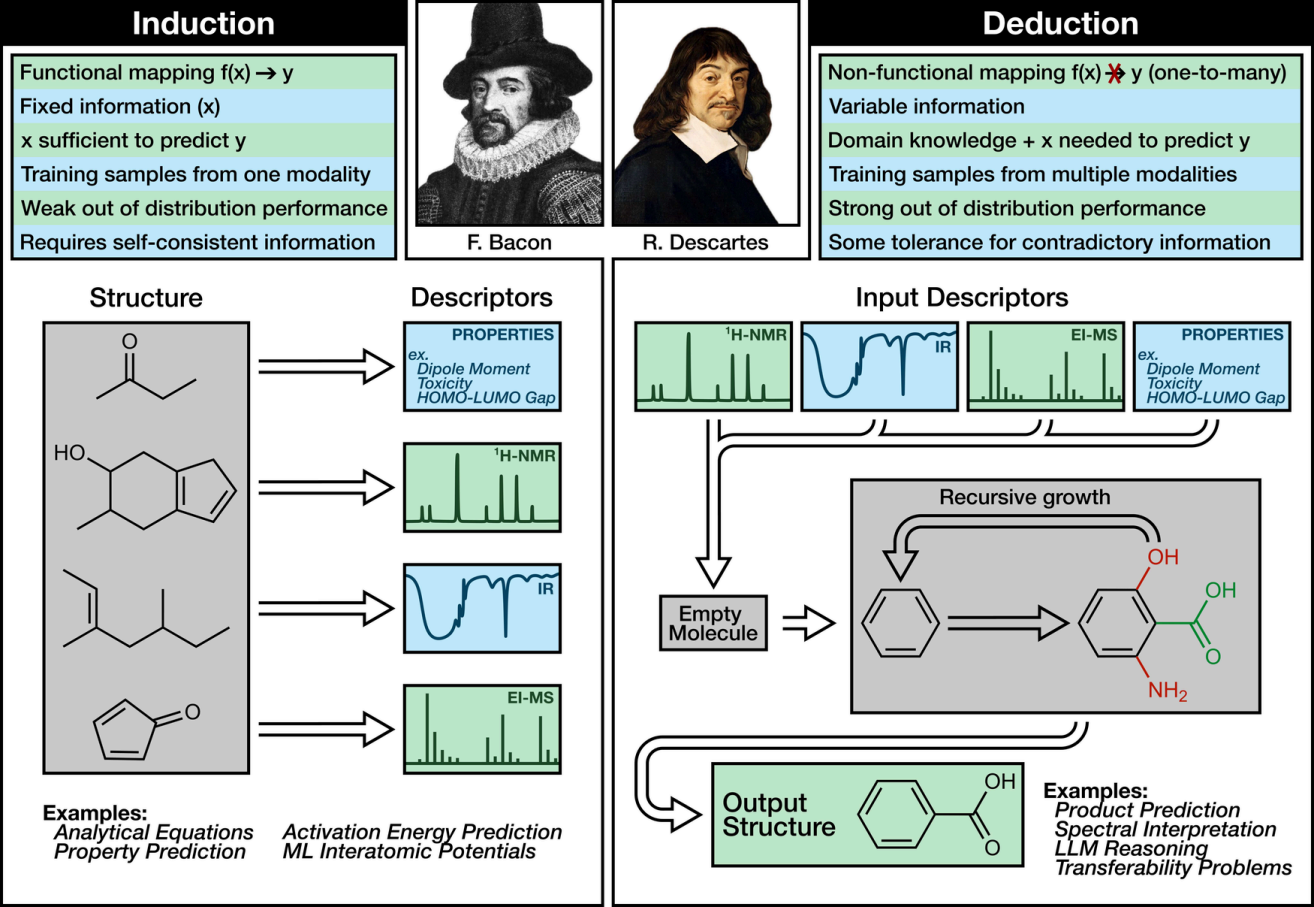
A conceptual overview of the deductive chemical reasoning paradigm compared to the inductive paradigm, illustrating their intellectual progenitors, key distinctions, conceptual schematics, and examples problems.

These increasingly complex problems often involve implicit assumptions in the output space which are difficult to quantify for ML methods. For example, models trained to propose novel structures for drug-like molecules that satisfy a certain property set also should yield output molecules that are (1) stable, (2) nontoxic, and (3) accessible via known synthesis routes—all unstated constraints that a human would intuitively understand but which a naive model could easily miss. Unsurprisingly, expert chemical intuition often remains the baseline for these deductive tasks.

### 6.1 Defining Chemical Reasoning: Unique Challenges

Chemical reasoning is defined here as the process of drawing mechanistically consistent conclusions about molecules, reactions, and materials by integrating diverse evidence (spectra, structures, conditions, kinetics, priors) under hard chemical constraints (stoichiometry, valence, conservation laws, thermodynamics etc.). Unlike purely inductive ML that maps *X* → *Y* by pattern matching, chemical reasoning often tackles inverse, one-to-many problems (e.g., spectrum→structure, properties→candidate molecules) and must justify why a proposed explanation satisfies the evidence and chemistry’s rules. The distinctive challenges inherent in chemistry reasoning can be systematically categorized into the following key factors:

- **Multiscale physics:** Real systems span quantum, atomistic, mesoscopic, and continuum scales with different formalisms and time scales. Coupling these consistently (and efficiently) is still an open problem; errors or approximations at one scale can cascade to others, complicating causal interpretation and design.^404^
- **Data sparsity, imbalance, and bias:** Reaction and property data remain sparse, heterogeneous, and biased toward successful outcomes, limiting causal learning and generalization despite efforts like the Open Reaction Database.^405^
- **Nonuniqueness (ill-posed inverse problems):** Multiple chemically distinct hypotheses can fit the same data, making solutions underdetermined and fragile without strong priors or multimodal constraints.^406^
- **Causal ambiguity and confounding:** Apparent yield or condition trends are often confounded by biases and sparse evidence, leaving reaction networks reliant on heuristics that blur causation and correlation.^407^

Despite the transformative advances of ML in predicting reaction outcomes and molecular properties, the field of computational chemistry remains fundamentally constrained by key challenges in chemical reasoning.

### 6.2 Inductive Chemical Reasoning

Inductive chemical reasoning, based on classical inductive reasoning as expounded by Francis Bacon’s *Novum Organum* ^1000^ in which broad generalizations are made from the patterns in data, has formed the foundation of most machine learning applications in chemistry. This paradigm operates under the principle of statistical pattern recognition to establish a direct, fixed mapping from the input representation *X* to a target output *Y*. For example, one common application of this approach is the Quantitative Structure–Activity/Property Relationship (QSAR/QSPR). Here, the input is a numerical encoding of a molecule or set of molecules (a learned numerical vector associated with a molecular structure), and the output is the predicted set of properties.^408^ Another common inductive problem is predicting reaction outcomes. In this workflow, the input *X* comprises the reactants, reagents and conditions, and the model ranks the candidate products *Y*.^366^

Despite achieving remarkable predictive accuracy, pure inductive models are fundamentally constrained by the nature of the data they are trained on. This constraint leads to challenges that compromise their utility as instruments of true scientific discovery. The most significant limitation is the vulnerability to Out-of-Distribution (OOD) generalization, or the ability to predict the properties or reactivity of molecules that are structurally or chemically distinct from the training set. Inductive models excel at interpolation (finding patterns within their defined chemical domain) but often fail at extrapolation. Often there are implicit biases in common chemical data sets toward synthesizability, stability, separability, or measurability of data that can significantly hinder inductive models. This failure leads to overconfident and inaccurate predictions when encountering novel or rare motifs. This issue is particularly pronounced when dealing with activity cliffs—molecules that are highly similar in structure but exhibit vastly different properties.^409^

### 6.3 Deductive Chemical Reasoning

While inductive models discover general trends from data sets for interpolative tasks, deductive models learn to theorize rules that must be imposed upon the provided input data. This deductive paradigm is conceptually inspired from the deductive reasoning process outlined by René Descartes^1001^ in the *Discourse on Method*, wherein knowledge proceeds from general axioms such as physical laws to specific observations. Though not an exact analogy, this reasoning process captures the spirit of problems containing underspecified input conditions or requiring extrapolative solutions. For example, an inductive EI-MS prediction model might learn that the presence of exactly two chlorine atoms in a molecular fragment splits the peak into a series of observed peaks with an approximate 9:6:1 ratio of intensities. A deductive model—trained to interpret EI-MS spectra—might observe a 9:6:1 ratio in the spectrum, theorize that this ratio corresponds to two chlorine atoms in the fragment, and impose this theory on the specific output prediction. In the latter case, there are likely several other conceivable fragmentation patterns that correspond to the 9:6:1 ratio observed in the spectrum, but which the model deems less likely from its training. Inductive reasoning is typically correct but ultimately unprovable. Deductive reasoning is valid to the extent that the proposed theory is correct. Because deductive models typically impose a theory in the process of procuring their output, they can often be trained to be interpretable and robust to missing or contradictory information. Accordingly, because modern LLMs are inherently inductive and were trained with large amounts of chemical data, it is retrospectively intuitive that they exhibit impressive performance on some common chemical tasks, even rivaling the performance of bespoke models on those tasks. In the following, we will discuss some examples of inductive and deductive chemical reasoning.

#### 6.3.1 Advancing Structural Generators

Molecular graph generation forms the core of structural generative modeling, translating atoms and bonds into learnable graph representations that preserve chemical validity (e.g., valency), which represent the general axioms of deductive reasoning. Over the past decade, increasingly capable architectures, ranging from variational and autoregressive models to flow, transformer, and diffusion frameworks, have expanded our ability to generate novel, synthesizable, and property-guided molecular graphs.

Early models established key strategies for the chemical validity and control. Junction tree and constraint-regularized VAEs constructed molecules from substructures while enforcing graphical rules.^106,410^ Flow-based models introduced invertible mappings with exact likelihoods: GraphNVP decomposed adjacency and node attributes, and MoFlow extended this to bond-first generation with validity corrections.^411,412^ Autoregressive approaches such as MolecularRNN and GraphAF combined sequential generation and reinforcement learning to improve valency and property optimization.^413,414^ Hierarchical and fragment-based strategies enabled scalable decoding at multiple resolutions.^415,416^

Transformer and language model paradigms broadened controllability and interpretation. MolGPT applied decoder-only transformers for conditional generation by properties or scaffolds,^417^ while MolGen integrated chemical feedback to reduce invalid “hallucinated” molecules.^418^ Recent “graph-to-structured-text” encodings let LLMs output coherent molecular representations in formats such as JSON or XML.^419^ Hybrid methods, including reinforcement learning with Transformer-GANs, have explored property-driven generation.^420^

Diffusion models now dominate discrete graph generation. DiGress introduced discrete noise processes and transformer conditioning for node and edge types.^421^ Conditional diffusion models further improved control and efficiency through guided ODE sampling.^422^ Joint 2D–3D diffusion coupled bonding graphs with spatial coordinates for complete molecule generation,^423^ while DMol accelerated the process with schedule-driven updates and ring compression.^424^ Constrained approaches such as CoCoGraph enforce chemical validity while matching property distributions of real compounds.^425^

Discrete flows and one-shot generators remain appealing for speed and tractability. GraphDF introduced modulo-shift latent variables to improve discrete sampling,^426^ while AMCG achieved fast atomic–molecular generation and property optimization.^427^ JTreeformer integrated latent diffusion into a graph transformer for efficient, structure-aware decoding,^428^ and MolSnap explored few-step inference with causality-aware variational mean flows.^429^

Recent work has also refined the chemical constraints and knowledge integration. Retrosynthetic building-block assembly enhances synthesizability and control,^430^ and knowledge-driven self-supervision improves substructure and spatial awareness.^431^ Generative flow networks extend this idea to atomic-level action spaces, pretraining on proxy rewards before goal-conditioned fine-tuning.^432^ Together, these advances illustrate the growing sophistication of molecular graph generation models in producing novel, unique, and synthesizable molecular graphs and herald advances in conditioning via multimodal input data.

#### 6.3.2 Multimodal Deduction

The maturation of multimodal learning in chemistry has transformed inverse design and structure elucidation from spectra or property constraints into tractable learning problems and thus a demonstration of deductive reasoning. Multimodal models learn to deduce outcomes from multiple different types of input data. Foundational surveys have outlined this progression and emphasized unified architectures capable of fusing infrared, Raman, mass spectrometry (MS), and nuclear magnetic resonance (NMR) data with properties and molecular representations.^403^ Within this context, deductive reasoning frameworks have clarified how such multimodal models operate on ill-posed inverse mappings that integrate incomplete, redundant, or conflicting evidence.^401^ The emergence of pretrained molecular language models such as Chemformer established generalizable sequence-to-sequence foundations for synthesis, translation, and optimization, enabling conditional generation across diverse chemical modalities that represent deductive reasoning.^383^

Early efforts toward multimodal structure generation employed probabilistic and representation learning strategies to bridge disparate data sources. Bimodal variational autoencoders paired tandem mass spectrometry with molecular structures, demonstrating that shared latent spaces could reconcile limited experimental data.^433^ Complementary studies unified SMILES and graph representations to enhance cross-format correspondence and improve property prediction tasks, foreshadowing subsequent multimodal encoders for generative applications.^434^ Conditional molecular design further advanced via prefix prompting mechanisms that allowed property or pocket information to modulate decoding while mitigating interference between condition types.^435^ In the spectroscopy domain, CMGNet exemplified this trend by combining ^13^C NMR spectra with formula and fragment constraints to propose candidate structures and assist in structural revision.^436^

The transition from conditional inference to direct end-to-end spectrum-to-structure generation marked a significant conceptual step. Hybrid convolutional–transformer frameworks achieved inference of molecular formulas and connectivities from routine one-dimensional ^1^H NMR and ^13^C NMR spectra, providing rapid and accurate structural predictions for small organic molecules.^437^ Transformer-based chemical language models subsequently replaced the rule-based CASE systems, enabling the direct translation of spectral signatures—including IR, UV-vis, and NMR—into candidate structures through learned sequence decoding.^438^ Deductive fusion networks extended these ideas by integrating multiple simulated and experimental spectra to identify reaction products under uncertain or incomplete information, generalizing effectively beyond the training regime.^401^

Recent advances in molecular foundation models have generalized this multimodal paradigm to a broader chemical context. Cross-modal pretraining frameworks such as MolFM aligned molecular structures, textual annotations, and knowledge graph embeddings through attention-based integration, capturing both local and global chemical relations for retrieval and transfer learning.^439^ More recent transformer baselines combined SMILES, two-dimensional graphs, and three-dimensional conformers within a single encoder to yield unified representations supporting conditional generation.^440^ Building upon these foundations, large-scale multimodal architectures have begun to connect spectra directly with three-dimensional geometry, albeit at this point for relatively simple molecules. MolSpectLLM unified experimental spectroscopy and 3D structure inference atop a 7-billion-parameter large language model, achieving competitive performance in spectra analysis and structure generation.^441^ Diffusion-based systems such as DiffSpectra further advanced this approach by conditioning an SE(3)-equivariant generator on multimodal spectral inputs to generate both two- and three-dimensional molecular structures, emphasizing the value of geometric inductive biases and pretrained spectral encoders.^442^

The emergence of dedicated benchmarks has facilitated evaluation of multimodal reasoning and structure generation. Tasks that treat structure elucidation as a “molecular puzzle” test whether large language models can integrate textual, numerical, and spectral cues within a coherent reasoning pipeline.^443^ In parallel, large property models have reframed continuous property vectors as conditioning tokens for generative molecular design, linking property prediction and structural inference within a single deductive interface.^444^ Complementary research has scaled deep learning interpretation of IR and Raman spectra for rapid characterization across molecular classes.^445^ Broader developments in 3D-aware multimodal LLMs for chemistry—including ChemMLLM, Chem3DLLM, and 3DMolFormer—illustrate convergent trends toward dual channel transformers capable of jointly processing textual, spectral, and structural inputs.^446–448^ Together, these works suggest that spectra informed, geometrically aware foundation models may soon enable unified deductive reasoning across more numerous modalities and scales. Future challenges in the field will have to apply these methods to more complex molecules, including natural products, and incorporate information from modern 2D and 3D NMR techniques.

### 6.4 Data and Problem Formulation for Chemical Reasoning

Existing, large-scale data sets in organic chemistry are useful for initial ML tasks but primarily focus on end point prediction and correlation.^202^ The design and structure of these data sets limit a model’s ability to perform true chemical reasoning by preventing models from learning the underlying mechanistic principles and causal structure of chemical transformations. To enable the next generation of AI to move from pattern recognition to genuine chemical reasoning, reasoning-oriented benchmarks must be created. This requires a shift from simple input/output data to data sets that explicitly encode mechanistic and causal information in a chemistry-aware fashion that learn the principles of chemical reactivity as part of the models. Recent efforts, including the development of more exhaustive schemas like the Open Reaction Database (ORD),^449^ represent critical steps toward proving the data to learn this mechanistic information.

### 6.5 Large Language Models for Chemistry Reasoning

Large language models (LLMs) constitute an interface between human chemists and the inductive and deductive paradigms outlined above. Trained predominantly as statistical next-token predictors on large corpora of scientific text, code, and tabular data, they are intrinsically inductive models, yet are routinely tasked with solving problems that chemists would describe as deductive. As outlined above, **Inductive** tasks require generalizing patterns from small tables or few-shot exemplars (e.g., substituent or homologous-series trends, simple condition–yield regularities, text mining), which show up in chemistry-focused benchmarks as extrapolation/judgment calls beyond rote recall.^212^ **Deductive** tasks apply codified rules to concrete cases (e.g., equilibrium, acid–base, thermodynamics, stoichiometry, spectroscopy) and are best handled when the model decomposes the problem into explicit steps and adheres to unit/constraint checks.^450^

Current approaches to chemistry reasoning with LLMs can be organized into three families: (1) direct LLM use, (2) LLM agents, and (3) task-specific LLM training. Direct LLM use treats the model as a standalone solver for textbook-style, well-posed problems (e.g., stoichiometry, equilibria, simple thermodynamics or spectroscopy questions). It relies on the model’s internal priors and chain-of-thought decomposition;^451^ performance improves with carefully crafted prompts, unit checks, and explicit step-by-step derivations. LLM agents decompose a task into substeps and then call external chemistry tools (Chemistry calculation tools, retrieving external chemistry domain knowledge, etc.) with intermediate verification. This reduces purely linguistic failure modes by grounding intermediate results, yet introduces orchestration complexity (tool selection/parametrization, error handling) and requires robust interfaces and guardrails.^452^ LLM training such as Supervised Fine-tuning (SFT)/Preference Learning/Reinforcement Learning (RL) or domain-adaptive pretraining, specializes models for chemistry tasks, aligning formats (e.g., dimensional analysis steps, mechanism justifications) and reducing style variance; it demands curated data, careful reward/label design.^128^

Empirically, LLMs perform best on well-posed, rule-driven deductive problems typical of college- and graduate-level benchmarks (e.g., GPQA, SciBench), especially when problems can be solved through transparent step-by-step derivations and consistency checks.^450,453^ Performance is mixed on inductive trend extrapolation in data-scarce settings that frequently occur in chemistry, as documented in chemistry-specific evaluations that separate recall from reasoned application.^212,443,454,455^

Several gaps limit the use of LLMs as full-fledged reasoning chemists: (i) evaluation coverage and process auditing: most QA suites report final answers but underweight decomposition quality and constraint satisfaction;^453^ (ii) mechanistic depth and extrapolation: models lack built-in physicochemical inductive bias and calibrated uncertainty, leading to confident but incorrect conclusions outside the training manifold.^454^ These gaps reflect core LLM limitations: probabilistic text generation without truth guarantees (hallucination), weak uncertainty calibration, limited hard-constraint enforcement across multistep derivations, and shallow treatment of physical priors, highlighting the need for process-aware evaluation and stronger constraint handling in future work.

### 6.6 Challenges and Opportunities

Chemistry operates within a constrained predictive space. Across nearly all scenarios, there are strict limits on feasible property values, atom types, permissible connections, possible reactions, and most other variables of chemical interest. This is in contrast to fields such as computer vision, where both the input and output domains are virtually unbounded. Within these constraints, chemistry served as a proving ground for architectural and conceptual advances in ML, offering a controlled environment in which methodological improvements could be benchmarked. These inherent boundaries demonstrate the distinction between the inductive and deductive paradigms. Computational chemistry occupies a unique position within ML research in the broader area of chemistry: it can evaluate emerging methods using real, physically grounded data under conditions that minimize confounding noise. As a result, such methods are more likely to succeed or fail on their intrinsic merits rather than on artifacts arising from the data complexity or ambiguity.

Despite this advantage, several data challenges persist, most notably (1) the scarcity of large-scale multimodal data sets encompassing high-quality and practically useful modalities and (2) the persistent disconnect between freely available and proprietary data sources in academia and industry. While substantial curation efforts have been made in recent years, still more effort is required to support the next frontier of deductive chemical models. Beyond the data, evaluation of the data presents another difficulty. Despite human experts often representing the current standard, few studies directly compare model outputs to those of expert analysis. While such comparisons are inherently complex and prone to bias, the performance gap between experts and models (if it exists at all) remains largely unknown and warrants an extensive study. Finally, because deductive models are structured around an interpretable reasoning process, these architectures could become more transparent with a minimal modification. Often, this opportunity has been overlooked. Building trust among experimental chemists, industrial practitioners, and machine learning researchers will likely depend on the degree to which models can articulate their decision pathways. Any effort to reveal the reasoning process should therefore be encouraged.

### 6.7 Chemistry Pushes AI Boundaries

Chemistry’s inverse problems and requirements for mechanistic reasoning (that is, tracing a model’s behavior to its internal components and parameter pathways) have driven a conceptual shift in AI from purely inductive pattern matching toward deductive inference under constraints. Unlike forward prediction tasks that map inputs to unique outputs (image classification, sentiment analysis), chemical reasoning routinely confronts one-to-many mappings where multiple plausible hypotheses explain the same observations. For example, spectral signatures can arise from structurally distinct isomers, synthetic targets can be reached through numerous disconnection strategies, and reaction outcomes depend on subtle changes in mechanistic pathways that are not shown in product structures alone. Addressing these ambiguities drove the development of multimodal fusion architectures that integrate heterogeneous evidence streams (NMR spectra, mass fragmentation patterns, elemental composition, literature priors etc.) not as mere feature concatenation of data but as logical constraints that jointly narrow hypothesis spaces. This paradigm has inspired deductive neural frameworks, constraint-satisfaction layers in generative models, and retrieval-augmented reasoning systems that ground predictions in structured domain knowledge rather than statistical correlation. Similarly, the demand for mechanistic interpretability, i.e. the understanding not just what will happen but why, through chemistry-aware models that consider electron flow, orbital interactions, kinetics, and thermodynamic driving forces has motivated attention-based explainability methods, causal representation learning, and physics-informed neural architectures that encode conservation laws and reaction principles as differentiable constraints. These advances, emerging from chemistry’s requirement to reason backward from effects to causes under incomplete information, now inform AI systems across scientific domains where inverse design, multihypothesis evaluation, and mechanistically grounded prediction are essential.

## 7 Agentic AI: Autonomous Chemical Discovery

### 7.1 A Definition of Agentic AI

Agentic AI systems in chemistry are autonomous or partially autonomous AI frameworks designed to reason, plan, and carry out complete scientific workflows within chemical research settings. In contrast to traditional AI tools that handle narrow predictive tasks, these systems demonstrate scientific agency, i.e., the capacity to independently formulate hypotheses, design and perform experiments (frequently in conjunction with robotic laboratories), interpret results, and continuously improve chemical models or synthesis pathways using feedback and new information.

Agentic AI in chemistry in the context of this Review thus denotes systems that not only assist but autonomously perform and optimize both the intellectual and experimental dimensions of chemical research. By uniting computational reasoning with laboratory automation, these systems operate within an integrated framework defined by autonomy, adaptive learning, and purposeful scientific exploration.

### 7.2 The Vision of Autonomous Chemistry

Modern chemical research includes an expanded design space that exceeds the capacity of traditional manual experimentation. Exploring this complexity demands automation, enabling continuous, data-driven workflows that integrate machine learning with robotic execution.^456^ Such autonomous or self-driving laboratories (SDLs) can iteratively design, perform, and analyze experiments, allowing algorithms to act as active scientific agents rather than as passive analytical tools.^457^ This is a very active and rapidly developing field, where many components need to interact seamlessly. Here, we focus on the AI components needed in agentic AI.

The necessity of automation extends beyond the efficiency. Manual experimentation is constrained by time, reproducibility, experimental error, and human bias in the experimental design. Autonomous systems help to overcome these limitations by operating continuously and systematically, generating high-quality, reproducible data sets that are essential for training reliable predictive models.^458^ In this way, automation enhances both the speed and reliability of discovery.

The potential impact of autonomous chemistry is transformative. By coupling AI reasoning with robotic precision, SDLs accelerate discovery cycles, reducing the time from hypothesis to validation from years to weeks.^459^ They also increase reproducibility through standardized digital protocols and consistent robotic execution. Cloud-connected and open-access laboratory infrastructures allow scientists worldwide to design and execute experiments remotely independent of local resources.

Ultimately, the vision of autonomous chemistry is not merely to mechanize the laboratory but to redefine the scientific process itself by creating a self-improving research ecosystem in which AI agents and automated platforms jointly drive hypothesis generation, experimentation, and knowledge creation.^456^

### 7.3 Components of Chemical AI Agents

Chemical AI agents operate at the intersection of computational reasoning and chemical experimentation, requiring specialized architectural components that distinguish them from those in pure AI domains. Unlike agents for text generation, game playing, or financial analysis, chemical agents must ensure chemical validity, enforce safety constraints, interface with physical laboratory equipment, and reason across multiple scales from single molecules to bulk materials.

#### 7.3.1 Domain-Specific Chemical Components

At the foundation of any chemical agent lies a *chemical knowledge base and constraint system* that encodes chemical information such as reaction rules, molecular graphs, thermodynamic and kinetic information, or potential safety hazards.^460^ This knowledge prevents the agent from proposing chemically impossible structures (e.g., pentavalent carbon) or dangerous reaction conditions (e.g., mixing of incompatible reagents). For example, Coscientist^461^ integrates safety checks that query chemical databases to flag hazardous procedures before execution, while ChemCrow^460^ validates molecular structures through RDKit cheminformatics tools at each planning step.

Equally critical is the *laboratory interface and protocol translation layer*, which converts abstract experimental plans into executable commands for robotic platforms, liquid handlers, and analytical instruments.^462^ A-Lab,^463^ for instance, translates high-level synthesis goals into robotic actions, including temperature control, reagent dispensing sequences, and measurement schedules while maintaining real-time communication with furnaces, balances, and diffractometers. This capability, absent in software-only agents, is essential for closing the loop between the computation and experimentation.

#### 7.3.2 Core Agentic Architecture

Building on these chemistry-specific foundations, chemical agents employ standard agentic components adapted to the scientific domain. A goal specification and planning engine decomposes high-level scientific objectives (e.g., “discover a catalyst with >90% yield for this reaction”) into testable hypotheses and experimental sequences. Modern systems implement this through LLM-based chain-of-thought reasoning,^460^ Monte Carlo tree search guided by domain heuristics,^464^ or reinforcement learning policies trained on experimental data sets.^463^ The planning engine must balance exploration (testing novel hypotheses) with exploitation (refining promising leads) under resource constraints—a classic multiarmed bandit problem in the context of expensive, time-consuming experiments.

The perception and feedback system monitors experimental outcomes through multimodal data streams: analytical measurements (NMR, mass spec, UV–vis), visual observation (reaction color changes, precipitate formation), and process parameters (temperature, pressure, pH).^465^ Advanced systems like LabOS^466^ employ video from augmented reality glasses to track human chemists’ actions, detect procedural errors (e.g., sterile technique violations), and provide real-time corrective feedback. This perception layer transforms raw sensor data into structured observations that update the agent’s internal models and beliefs about the chemical system under study.

A *learning and adaptation module* enables chemical agents to improve over time through multiple mechanisms. Online model updating refines predictive models (reaction yield, property prediction) as new experimental data accumulates, enabling increasingly accurate forecasting Wei et al.^467^ Policy learning via reinforcement learning optimizes experimental strategies—which experiments to run next, how to adjust parameters in response to failures—using reward signals derived from experimental success.^464,468^ Meta-learning enables agents to transfer knowledge across related tasks, such as applying lessons from one catalyst optimization campaign to a structurally similar system.

#### 7.3.3 Human–AI Collaboration and Oversight

Chemical agents require transparent *communication and explanation interfaces* to maintain human oversight and trust. Agents must explain their reasoning (“I propose this experiment because simulations predict 85% yield, and literature precedent supports stability”), justify safety decisions, and accept human intervention when scientists identify flaws or ethical concerns. Multiagent systems like those described by Ünlü et al.^469^ maintain full provenance tracking by recording which agent proposed each molecule, what reasoning supported the proposal, and how the design evolved with the goal of ensuring auditability and interpretability throughout the discovery process. This transparency is essential not only for scientific reproducibility but also for responsible deployment of autonomous systems in laboratory environments where errors can have physical consequences. To teach agents to use chemical tools or follow complex experimental procedures, high-quality instructions play a crucial role in reinforcement learning, as synthesized in a recent work.^470^

Unlike agents in domains such as text, games, or logistics, chemical agents must handle complex, high-dimensional chemical spaces, where molecules, reactions, and materials obey strict physical and chemical laws. This necessitates domain-specific Knowledge Base and Memory, including reaction rules, molecular graphs, and thermodynamic constraints, to ensure that proposed experiments or molecules are chemically valid.^460^ They also require integration with laboratory hardware—automated synthesis platforms, robotic liquid handlers, or spectroscopy instruments—to physically execute chemical reactions, a capability unnecessary in most software-only domains.^462^ Furthermore, safety, hazard assessment, and chemical feasibility modules are crucial, as incorrect actions can produce dangerous reactions or compounds. These components collectively make chemical AI agents uniquely bridged between computation, experimentation, and chemical law, which is not typically required in other AI domains like finance, language, or games.

### 7.4 Task Landscape for Chemical Agents

AI agents are increasingly capable of handling complex, multistep chemistry research tasks that go far beyond simple data analysis or literature searches. As summarized in Table 6, these advanced applications span a wide spectrum of the scientific process, ranging from hypothesis creation and simulation to autonomous experimental planning.

**Table 6:** Summary of Work on Agentic AI for Chemistry^a^

Task	Tools	Reference	Feedback	Modalities	Benchmarks	Human-in-the-Loop
Literature Review	(1) Web and Literature Search	^456^	N/A	Text Spectra, Images	x	x
		^471^	N/A	Text	x	x
		^458^	N/A	Text	x	x
Hypothesis Creation	(1) Web and Literature Search	^472^	Benchmark Success/Failure	Text Tabular Data	GPQA Diamond	√
	(2) Code Execution					
	(3) Domain DBs	^473^	Automatic Link-Prediction Ranking Scores	Text Knowledge Graph	Custom Tasks	x
	(4) Knowledge Graphs					
Chemical Innovation	(1) RDKit and Property Calc.	^474^	(1) Property Scores (LogP, etc.)	Text Numeric Props	Custom Tasks	x
	(2) Similarity/Filtering Tools		(2) Heuristic Constraints (Valid, Uniq., Sim.)			
	(3) Docking and Property Models	^469^	(1) Docking/Simulation Scores	Text Numeric Props	Custom Tasks	x
	(4) Chem. DBs (UniProt, etc.)		(2) Property Scores			
		^475^	(1) Binding/Docking Scores	Text Numeric Props	OpenTargets Protein-Target	x
			(2) Drug-Likeness, SA, Novelty, Diversity			
Simulation	(1) QC Simulators (DFT)	^476^	(1) Simulation Scores	Text Numeric Props Spectra	Quantum Course (6 Problems)	x
	(2) Code Execution		(2) Benchmark Success/Failure			
	(3) ML Models for Screening	^477^	(1) DO Score Labels	Text Numeric Props	DO Challenge VS Benchmark	x
	(4) API		(2) Time/Resources			
Experiments Planning	(1) Web/Docs Tools	^466^	(1) Exp. Measurements (Outcomes, Sensors)	Text Image/Video Sensor Data	Custom Tasks	√
	(2) Lab Remote Platforms					
	(3) Safety Checkers		(2) Human Feedback (Language, Gestures)			
	(4) Chem. DBs					
	(5) Computer Vision System	^461^	(1) Exp. Measurements (Yields, Spectra)	Text Spectra Numeric Props	Custom Tasks	x
	(6) Real-Time Control					
			(2) Simulation Scores			
			(3) Benchmark Success/Failure			
		^463^	(1) Exp. Measurements (XRD, Phases)	Text XRD Spectra Numeric Props	Custom Tasks	x
			(2) Simulation/Model Scores			
		^478^	(1) Route Feasibility	Text	Constrained-Retro Benchmark	x
			(2) Safety Constraints (E.g., Carcin., Pyro.)			
		^465^	(1) Exp. Measurements	Text Image/Video Numeric Props	x	x
			(2) Vision-Based States: (Turbidity, Solids)			

a We categorize existing work by task type, available tools for agents, feedback sources, data modalities, benchmark usage, and the presence of human involvement. A √ indicates human-in-the-loop supervision, whereas an *x* denotes the absence of human involvement or benchmark evaluation.

#### 7.4.1 Agents for Automated Literature Review

Recent work shows agentic AI systems—typically LLM-centered multiagent frameworks or hierarchical agent stacks—are already being applied to automate literature-centric tasks in chemistry, such as paper discovery/scraping, extraction of experimental conditions, hypothesis generation from corpora, and feeding results into experimental planning pipelines. For example, Coscientist^461^ demonstrated the integration of LLMs with web and document search, tool use, and reasoning to consult the literature when planning chemical experiments autonomously. Similarly, ChemCrow^479^ augments LLMs with chemistry-specific tools and databases, enabling automated consultation of literature and chemical knowledge bases when performing synthesis planning or answering chemistry questions. Reviews and surveys^458,480^ further describe agentic pipelines that combine retrieval, structured information extraction, and iterative reasoning to generate literature summaries and candidate hypotheses from large corpora. Other recent work focuses more explicitly on automated extraction and structured knowledge construction from papers. For example, LLM-driven agent pipelines have been developed to extract material properties and experimental parameters from thousands of full-text articles, converting unstructured literature into machine-readable data sets for downstream modeling and discovery.^481^ In parallel, multiagent frameworks such as LatteReview automate systematic review workflows by coordinating specialized agents for paper screening, relevance scoring, and structured data extraction across large literature collections.^482^ Multiagent robotic chemists^483^ and domain-specific agent systems for computational chemistry^476^ extend these ideas by closing the loop: literature mining informs experiment design or computation, results are ingested back into the agent, and the agent refines its next literature queries and plans. Related hierarchical architectures, such as ChemHAS, further demonstrate how agent stacks can coordinate tool use and reasoning across chemistry workflows, improving both literature-derived insights and downstream task performance.^484^ Across these works, the recurring strengths are speed, ability to synthesize heterogeneous sources, and tighter integration with downstream automation; recurring challenges are ensuring provenance, extraction accuracy (especially for numerical experimental parameters), and robust evaluation metrics for automated reviews.

#### 7.4.2 Agents for Hypothesis Creation

Recent work shows agentic AI has moved from proof-of-concept assistants to systems that can generate, critique, prioritize, and sometimes experimentally validate hypotheses in chemistry and adjacent domains. Large surveys and reviews summarize architectures (single LLM agents, multiagent teams, knowledge-graph + tool stacks) and show hypothesis generation is a core capability of these systems, often coupled to experiment design and lab automation pipelines.^485^ Case studies and system papers demonstrate end-to-end pipelines: classical “self-driving labs” that close the loop between hypothesis proposal and automated experiments (accelerating materials discovery), LLM-guided frameworks for proposing testable chemical hypotheses and parameter searches, and chemistry-specific workflows that combine sampling strategies, knowledge graphs, and active validation.^463^ Several recent systems illustrate the diversity of agentic hypothesis-generation pipelines. For example, the ChemCrow framework augments LLMs with chemistry tools (reaction prediction, molecular property estimation, and literature retrieval) to enable hypothesis-driven reasoning about chemical reactions and synthesis planning.^479^ The MOOSE-Chem family of systems explores whether LLM agents can rediscover scientific hypotheses from chemistry corpora and rank them using simulated experimental feedback, demonstrating that agentic reasoning can recover hidden scientific relationships from literature-scale data sets.^486^ Closed-loop laboratory systems further demonstrate hypothesis generation integrated with experimentation. Platforms such as ChemOS orchestrate autonomous experimental campaigns in self-driving laboratories, iteratively proposing candidate molecules or reaction conditions using predictive models and validating them through robotic experimentation.^487^ Industry and large-lab setups (Google’s AI coscientist and related agentic research prototypes) show that multiagent LLM setups can propose novel hypotheses and research plans, though authors consistently note limitations in robustness, evaluation metrics, domain grounding, and human oversight needs.^472^ Across the literature, the main open problems are (1) reliable quantification/validation of AI-generated hypotheses, (2) safe integration with real laboratories and instruments, and (3) building chemistry-aware scientific foundation models or knowledge representations to reduce hallucination and improve testability.^473^

#### 7.4.3 Agents for Chemical Innovation

Recent research on Agentic AI for chemical innovation demonstrates fast progress in autonomous discovery workflows. The AgentDrug^474^ framework introduces nested feedback loops between large language models (LLMs) and cheminformatics tools to ensure molecular validity and guide optimization objectives efficiently, reducing invalid molecule generation.^469^ proposes a hierarchical multiagent system performing de novo design, docking, and ranking while maintaining full transparency of reasoning through provenance tracking; multiagent setups achieved a 31% improvement in binding affinity relative to baseline models. LIDDiA^475^ highlights natural-language-driven planning via modular, tool-using agents to explore vast chemical spaces for novel compounds. Subsequent work has refined tool integration and search strategies. CheMatAgent^488^ addresses the limitations of earlier agents by synergistically coupling an LLM with 137 Python-wrapped chemistry tools spanning basic information retrieval through complex reaction prediction, governed by a novel Hierarchical Evolutionary Monte Carlo Tree Search (HE-MCTS) framework that independently optimizes tool-planning paths and execution parameters, outperforming GPT-4o on domain-specific benchmarks. In the domain of property estimation, MolAgent^489^ provides a system-agnostic agentic framework for high-fidelity molecular property modeling in early stage drug design, autonomously implementing expert-level classification and regression pipelines with automated feature engineering and ensemble model selection. Agentic systems are also entering real-world industrial deployment. AstraZeneca’s ChatInvent^490^ represents a rare documented case of an agentic system integrated directly into an active pharmaceutical pipeline, evolving from a single proof-of-concept agent into a scalable multiagent architecture supporting molecular design and synthesis planning. Similarly, the ChemAgents system^483^ demonstrates a hierarchical multiagent robotic AI chemist built on an on-board Llama-3.1–70B LLM, coordinating role-specific agents—a Literature Reader, Experiment Designer, Computation Performer, and Robot Operator—to execute complex multistep experiments with minimal human intervention. The DO Challenge benchmark^477^ provides a standardized evaluation for agent systems in virtual screening, showcasing the “Deep Thought” multiagent system that autonomously executes screening strategies and approaches human-level performance in compound discovery. Collectively, these studies signal a paradigm shift toward self-improving, collaborative AI chemists capable of generating, evaluating, and optimizing molecules at scale through adaptive, transparent, and multiobjective frameworks for de novo design and screening automation.

#### 7.4.4 Agents for Molecular Simulation

Recent research on agentic AI in chemistry reveals a rapidly growing ecosystem of autonomous systems capable of simulating, monitoring, and optimizing chemical processes. Wei et al.^467^ outline frameworks where learning-enabled agents autonomously propose and validate reaction mechanisms by running molecular simulations that model reaction kinetics and thermodynamics. The El Agente Q^476^ demonstrates natural language-driven execution of quantum chemistry computations, where agents autonomously plan and carry out multistep simulations with minimal human guidance. FlowER generative AI,^491^ predicts chemical reaction pathways by conserving fundamental chemical principles, showing early success at mapping reactivity and validating proposed mechanisms. MDCrow^492^ extends agentic automation specifically to molecular dynamics workflows, using chain-of-thought reasoning over 40 expert-designed tools to handle file processing, simulation setup, output analysis, and literature retrieval, demonstrating robust performance across 25 tasks of varying complexity. At a higher level of abstraction, ChemGraph^493^ combines graph neural-network-based foundation models with LLMs to streamline and automate atomistic simulations and materials science workflows, providing an intuitive natural language interface that lowers the barrier of expert knowledge traditionally required for setup, execution, and validation. Autonomous laboratory platforms such as A-Lab^463^ integrate AI, robotics, and machine learning to continuously tune reaction conditions and optimize synthesis routes in real time. Together, these studies signal a shift toward self-guided AI chemists capable of seamlessly executing simulation, design, and optimization loops to accelerate the discovery.

#### 7.4.5 Agents for Experiment Planning

Recent work shows agentic/LLM-driven approaches are being integrated with automated synthesis planning and self-driving laboratories, while complementary ML and computer-vision methods enable real-time reaction monitoring. Notable themes across the papers are (1) LLMs and agentic architectures can autonomously propose experiments and orchestration strategies (demonstrated end-to-end in a GPT-4 driven system that planned and executed experiments).^461^ (2) Reviews of self-driving laboratories summarize the closed-loop DMTA (design–make–test–analyze) workflows, hardware/software stacks, and open challenges in robustness, data standards, and human–AI interfaces.^456^ (3) Automated synthesis/retrosynthesis modeling has matured from rule-based and template systems toward deep-learning and explainable frameworks that explicitly model multistep planning and provide interpretable actions for chemists.^494^ (4) New “agentic” LLM frameworks for constrained retrosynthesis: agents can evaluate, requery tools, and iterate on routes under constraints (cost, availability, safety), improving practical usability for experimental planning.^478^ (5) For real-time reaction monitoring, computer-vision and online spectroscopy + ML pipelines enable automated detection of physical state changes and time-series prediction of yields or mechanism indicators that are essential inputs for autonomous decision loops in robotic platforms.^465^

### 7.5 Feedback Loops

#### 7.5.1 Why Autonomous Chemistry Agents Need Feedback

Autonomous chemistry agents operate across sparse, noisy, and multimodal evidence (text, spectra, structures, protocols, lab videos) where purely linguistic priors are not suitable. Reliable feedback includes physics-/simulator-grounded scores, factuality checks against literature, and real-world procedural verification such as anchors hypotheses, prunes searches, and calibrated uncertainty. Beyond digital planning, modern systems increasingly require embodied/operational feedback in the wet lab: perception of human actions, error detection, and step-level conformance to protocols. LabOS^466^ crystallizes this need by showing that an AI coscientist must see what human scientists see (egocentric video), compare against gold-standard steps, and signal deviations in real time; only then can planning be safely closed with execution.

#### 7.5.2 How Current Feedback Is Constructed and Sent to the Agent

Current feedback mechanisms can be divided into several categories:

(1) Physics- and simulation-based feedback transforms atomistic evaluations such as adsorption energies, reaction barriers, and molecular stability into quantitative reward functions that guide exploration and prune unproductive branches in the search space.^464^ A complementary line of work uses simulated experimental proxies in place of real wet-lab results: MOOSE-Chem3 ^495^ introduces a domain-grounded simulator (CSX-Sim) that models hypothesis performance as a function of conceptual similarity to a hidden ground truth, perturbed by noise calibrated against 124 experimentally validated outcomes from the published literature.
(2) Policy-learning feedback encodes task success as delayed, session-level rewards (for example, PPO-style objectives) that enhance long-horizon decision-making and tool-use strategies, as demonstrated in paper search agents such as PaSa.^468^
(3) Human-AI procedural feedback in the lab (embodied loop). LabOS^466^ streams 5–10 s egocentric video segments from XR glasses to a lab-specialized VLM; the server returns structured JSON containing step recognition, error labels (e.g., sterile breach, timing mismatch), and next-action suggestions. This becomes real-time, on-bench feedback (visual/audio prompts) that validates adherence to protocol and documents deviations for reproducibility.

The different forms of feedback collectively guide the agent to explore promising regions or prune unproductive paths, enhance policy improvement under sparse rewards, and strengthen the operational robustness and safety.

### 7.6 Challenges and Open Problems

#### 7.6.1 Roadmap for Chemical Agents

Advancing chemical agents from current proof-of-concept systems to trusted scientific tools will require progress along five axes: (1) *agentic benchmarks* that evaluate full decision cycles—hypothesis quality, sample efficiency, and safety compliance—beyond isolated task accuracy;^477,478^ (2) *uncertainty-native planning*, where calibrated confidence is embedded directly into the agent’s action-selection loop rather than applied posthoc; (3) *safety as a first-class constraint*, with verifiable hazard-rejection layers and auditable reasoning traces evaluated by standardized metrics before deployment;^455^ (4) *modular, interoperable architectures* that allow planners, safety checkers, and robotic interfaces to be shared across laboratories via open-source frameworks; and (5) *equitable access* through open hardware standards, cloud-accessible platforms, and shared data repositories, ensuring that agentic chemistry benefits the broader scientific community.

Translating this roadmap into practice, however, requires addressing several core challenges that continue to constrain the widespread adoption and scientific reliability of agentic and autonomous systems in chemistry. Below, we examine five such challenges, spanning data infrastructure, reasoning integration, model reliability, hardware robustness, and safety, that must be overcome to close the gap between current proof-of-concept agents and dependable scientific tools.

- **Data standardization and interoperability**: Most chemical data remain fragmented across incompatible formats, lab notebooks, and unstructured literature, which limits model training and reproducibility. Developing unified ontologies and open repositories is essential for cross-lab collaboration and agent interoperability.^496^
- **Reasoning–experiment integration**: Current self-driving laboratories (SDLs) excel at optimization but lack scientific reasoning. Coupling LLM-based hypothesis generation and planning with physical experimentation remains an open problem.^497^ Achieving closed-loop systems that merge symbolic reasoning, machine learning, and robotics would mark a critical step toward true agentic discovery.
- **Model reliability and uncertainty**: Machine-learning models often perform well within trained regimes but extrapolate poorly in unexplored chemical spaces. For agentic systems, this is especially consequential: an autonomous agent that plans multistep experimental campaigns must judge when its own predictive models are trustworthy enough to commit real laboratory resources—a wrong decision can waste hours of robotic synthesis time or, worse, trigger hazardous conditions.^456^ Unlike a static prediction tool, an agent continuously acts on its model outputs (selecting the next experiment, adjusting reaction parameters, deciding when to explore vs exploit),^498–501^ so poorly calibrated confidence directly degrades the quality of the entire decision loop. Mitigation pathways within the agentic framework include equipping the planning engine with uncertainty-aware acquisition functions, such as Bayesian optimization^502–504^ or ensemble-disagreement criteria,^505^ that allow the agent to autonomously identify and prioritize high-information experiments while avoiding regions where its models are unreliable. Progress can be measured at the system level: the number of autonomous experimental cycles needed to reach a target objective, the fraction of agent-proposed experiments that yield actionable results, and the reliability with which the agent’s confidence estimates predict actual experimental outcomes. Robust uncertainty quantification and active-learning strategies are required to ensure reliable decision-making during automated exploration.
- **Hardware and operational robustness**: Automated platforms face persistent mechanical and calibration issues that compromise reproducibility and scale.^506^ Developing modular, low-cost, and fault-tolerant robotic systems remains a difficult engineering challenge, particularly for resource-limited laboratories.
- **Safety and ethical considerations.** As AI agents assume increasing autonomy in experimental design, ensuring interpretability, auditability, and ethical compliance becomes vital. Chemistry poses unique safety risks: an agent’s erroneous proposal can lead to hazardous reactions, toxic byproduct release, or misuse of dual-use synthesis knowledge. Zhou et al.^455^ recently reported that even the most advanced LLMs, such as GPT-4o and DeepSeek-R1, often fail to identify scientific laboratory hazards, achieving accuracies below 75%. Current mitigation efforts include multilayer safety-checking modules that cross-reference proposals against hazard databases before execution,^460,461^ provenance tracking that records the full reasoning chain behind each experimental decision,^469^ and human-in-the-loop checkpoints at critical decision nodes. Measuring progress in this area requires developing standardized safety benchmarks—for instance, test suites of known hazardous proposals that agents must reliably reject—as well as auditing metrics such as the completeness of reasoning traces and the rate of flagged interventions during autonomous operation.
- **Equity and accessibility**: The promise of democratized science depends on equitable access to automation infrastructure and shared data ecosystems. Without open-source agent frameworks, cloud-accessible robotic platforms, and globally shared benchmark data sets, agentic chemistry risks reinforcing existing disparities rather than alleviating them. A practical roadmap includes establishing community-driven benchmark suites (analogous to ImageNet^237^ or MoleculeNet^202^ for predictive tasks) specifically designed to evaluate agentic capabilities—covering hypothesis quality, experimental efficiency, and safety compliance—alongside investment in open hardware standards and remote-access laboratory networks.

### 7.7 Chemistry Pushes AI Boundaries

The iterative, decision-intensive nature of chemical experimentation has driven the development of autonomous agentic AI frameworks that integrate planning, execution, and adaptive learning in closed-loop workflows. Unlike domains where AI systems operate purely digitally (recommendation systems, language translation, game playing), chemical discovery requires embodied agency—the capacity to interface with physical laboratory instrumentation, execute multistep experimental protocols under safety constraints, interpret multimodal sensor feedback (spectroscopy, imaging, process telemetry), and dynamically replan based on real-world outcomes that may contradict computational predictions. This requirement has motivated architectures that couple high-level reasoning engines (LLM-based planning, Monte Carlo search, reinforcement learning policies) with low-level control systems (robotic manipulation, temperature regulation, reagent dispensing), safety verification layers (hazard checking, constraint satisfaction), and online learning modules that update models as experiments complete. The requirement to balance autonomous operation with human oversight, maintain interpretability and auditability throughout decision chains, and operate reliably despite mechanical failures and sensor noise has made chemistry a proving ground for trustworthy, safety-critical agentic AI. These design principles—transparent reasoning traces, human-in-the-loop intervention points, and robust handling of real-world uncertainty—now inform autonomous systems in other physical domains where AI agents must act responsibly in environments with irreversible consequences, including materials synthesis, pharmaceutical manufacturing, and laboratory automation across the sciences.

## 8 Conclusions and Future Perspectives

### 8.1 Summary of Progress

This review examines how AI methods are beginning to address long-standing challenges in organic chemistry, how new AI methods are developed to respond to these challenges, and what open questions remain for future work. Community efforts to standardize experimental records and curate multimodal data sets are gradually improving data quality.^1,3^ Meanwhile, imbalance-aware training objectives address the challenge of learning from sparse, unevenly distributed chemical data.^221^ Representation learning now spans SMILES strings, molecular graphs, 3D conformers, spectra, and natural language descriptions. Data augmentation strategies help address limited training data for rare but valuable molecular properties.

Reaction prediction has moved beyond expert systems to neural-symbolic hybrids capable of planning at scale, while chemically grounded language and graph models increasingly reason across forward and retrosynthetic tasks.^366,368^ Multimodal foundation models push chemical reasoning toward deductive settings by aligning spectra, structure, and narrative evidence.^441^ Agentic AI closes the loop by coupling these predictors with planning engines, robotic execution, and safety-aware feedback, demonstrating autonomous end-to-end discovery workflows.^460,461^

### 8.2 Persistent Challenges

Data scarcity and selection bias remain the primary bottlenecks. Reaction data sets systematically exclude negative results, failed attempts, and contextual metadata (reagent purification, mixing protocols, temperature ramps), creating severe selection bias.^13,15^ Models trained on biased data fail to generalize: they produce confident but incorrect predictions for novel molecular structures or property values not well represented in training. Standard uncertainty estimation methods (ensembles, Gaussian processes) cannot identify when predictions are unreliable because of missing training examples.

The reproducibility of legacy data remains limited. Many reported results depend on proprietary data sets, unreported data splits, or expensive pretraining that cannot be replicated by independent groups. Benchmark data sets contain temporal leakage (test molecules synthesized after training data collection) and duplicate structures that artificially inflate performance metrics. High benchmark accuracy does not guarantee experimental success. Models that perform well on benchmarks often fail in the laboratory due to sensitivity to unmeasured reaction conditions or unusual substrate combinations.

For routine reactions, traditional retrosynthesis currently outperforms the neural models. Human experts handle stereochemistry and protection group strategies more reliably. Most academic laboratories cannot afford to train foundation models from scratch and must rely on transfer learning or active learning instead.

### 8.3 Emerging Directions

Multimodal foundation models that combine SMILES, spectra, and text could improve predictions with a limited amount of training data. However, current architectures generalize poorly to molecules or properties outside their pretraining distribution.^439,441^ Scaling laws and sample efficiency trade-offs for molecular data remain poorly characterized compared to text or images.

Automated synthesis platforms combine robotics, analytics, and AI planning but face limitations in hardware reliability, reagent handling, and safety.^466^ Most demonstrations focus on well-studied reactions rather than pushing into new reaction space. Scaling to broader reaction types requires better sensor integration, feedback control, and error handling.

Evaluation benchmarks need to be reformulated. Current metrics (top-k accuracy and RMSE) do not capture critical desiderata: calibration under distribution shift, sensitivity to missing metadata, or deductive reasoning over reaction mechanisms. Developing benchmarks that test out-of-distribution generalization, mechanistic consistency, and uncertainty quantification will clarify which architectural choices improve robustness.^485^

Finally, incorporating physical constraints (mass balance, thermodynamic feasibility) and sustainability metrics (atom economy, E-factor, toxicity) as differentiable objectives or hard constraints could steer generative models toward synthesizable, green chemistry, though multiobjective optimization with heterogeneous constraints remains computationally expensive and underexplored.

### 8.4 Toward Integrated Chemical Intelligence

Realizing the potential of AI in organic chemistry requires sustained collaboration between chemists, computer scientists, and other domain experts. Shared ontologies, open data infrastructures, and reproducible software are prerequisites for exchanging hypotheses, code, and experimental protocols across institutions.^458^ Governance frameworks that mandate transparent reasoning traces, enforce safety protocols, and ensure responsible use of AI to preserve human oversight will be essential as autonomous systems become more prevalent. AI tools should augment and enable rather than replace chemical expertise—surfacing design spaces that warrant investigation, accelerating hypothesis testing, and allowing chemists to focus on problems requiring creativity, mechanistic insight, and experimental judgment.^461^ Success will depend on maintaining high standards for validation, acknowledging when traditional methods remain superior, and cultivating the interdisciplinary expertise required to critically evaluate and responsibly deploy these emerging technologies.

## Abbreviations

Autoencoder: A machine learning framework consisting of an encoder that maps input data to a latent feature vector (of dimension *k*, e.g., 64, 128, or 256) and a decoder that reconstructs the input from this representation. The latent vectors can be used in downstream applications.
Autoregressive models: A class of ML models that generate outputs sequentially, where each new element is predicted based on the elements generated before it. For example, in language models it predicts the next word given the previous words.
Auxiliary tokens: Tokens that mark structural information, such as the start or end of a sequence (<BOS> or < EOS>), or indicate specific tasks (e.g., < CLS>).
Bayesian meta-learning (optimization): A framework that provides a recipe for making task predictions using uncertainty variables and combining information from observed low-data settings to guide future experiments in search of optimal reaction conditions.^205^
Bimodal variational autoencoder (BiVAE): A type of model capable of learning from data with missing annotations during the supervised training and test phases.^433^
Classifier-guided diffusion (CGD): A method that involves enhancing the generation process of diffusion models with guidance from the gradients of a classifier.^302^
Conditional diffusion model: A type of model trained to generate *X* with specific values *Y* by directly conditioning on target property vectors.
Constraint-satisfaction: The process of solving a problem by meeting a specific set of limitations or requirements. Constraint-satisfaction layers or problems are often represented as constraint graphs, where nodes represent variables and edges represent constraints between them.
Contrastive SSL: A method where a model learns representations without labeled data by comparing examples. It learns to maximize the similarity between positive samples (e.g., molecules augmented from the same original molecule) and minimize the similarities between negative samples (e.g., molecules from different molecules).
Convolutional neural network (CNN): A type of neural network designed to detect local patterns in structured data, such as images or grids. They use small filters that slide across the input to identify features like edges, shapes, or repeating structures. By stacking multiple layers, CNNs learn increasingly complex features. CNNs are often used to recognize and segment molecular structures from chemical documentation.
Cost-aware (graph search, metrics): A process in which a model reasons over a graph’s nodes and edges in a manner that minimizes costs such as time, energy, and money.
Decoder: In the framework of Autoencoder and Variational Autoencoder, it is a neural network that takes the encoded vectors from the encoder layer and generates an output.
Deep network: A type of neural network that stacks multiple input and output layers together, increasing structural representation within input and output graphs.
Diffusion models: A subclass of deep models for generation, most commonly through a transformer backbone. Diffusion models conduct generation by adding noise to clean input data, then attempting to denoise the result while estimating a score function.
Discrete flows models (DFM): A discrete data generative model built around a probability flow that interpolates from noise to data.
Encoder: A neural network that processes and encodes input information into a series of numerical representations (vectors) that capture its contextual information.
Fine-tuning: The process of taking a pretrained model and further training it on a smaller, task-specific data set to adapt it to a particular application or domain. Fine-tuning adds additional neural layers on top of the existing model.
Flow-based models: A type of generative models that learn to transform a simple probability distribution (such as Gaussian noise) into complex data through a series of reversible and invertible mathematical transformations. In chemistry, flow-based models can be used to generate molecular structures or model reaction transformations by mapping simple latent variables to valid molecular graphs or SMILES strings.
Foundation models: A base model, trained on a broad data set, allowing it to be tailored to a variety of downstream tasks.
Generative adversarial network (GAN): A class of generative models that learn to create new data samples that resemble patterns from the training data. These models pair a generator, which learns to generate from training data, with a discriminator, which tries to distinguish real data from generated data. Through this adversarial process, the generator gradually learns to produce data that closely resembles the real data set, such as augmentations of molecular structures.
Generative learning: A model that emulates the structure and characteristics of input data in order to generate derived synthetic content.
Gradient-based optimization: A mathematical technique used to find the minimum or maximum of a function by leveraging the gradient (i.e., derivative), which represents the direction of the steepest ascent or descent.
Graph diffusion transformers: A class of models that combine diffusion generative models with Transformer architectures to generate or model graph-structured data. The diffusion process gradually transforms noise into a valid graph, while the Transformer captures complex relationships between nodes and edges. In chemistry, these models can be used to generate molecular graphs, where atoms are nodes and bonds are edges, enabling the design of new molecules with desired properties.
Graph neural network (GNN): A type of neural network designed to learn from graph-structured data, where nodes represent entities and edges represent relationships between them. The model updates each node’s representation by aggregating information from its neighboring nodes. In chemistry, molecules can be represented as graphs where atoms are nodes and chemical bonds are edges, allowing GNNs to learn relationships between molecular structure and properties such as reactivity, toxicity, or binding affinity.
Heuristic algorithms: An algorithm that provides an answer that is close to the correct answer. Heuristic algorithms function very quickly and allow for approximate predictions on complex data sets.
Hybrid convolutional–transformer frameworks: A framework that combines Convolutional Neural Networks with a Transformer model, enabling image recognition and a self-attention mechanism to capture image information in a cohesive framework.
Hypergraph NN (HNN): A type of neural network that uses a hypergraph structure for its data storage. Hypergraphs store information using hyperedges, which allow higher order data connectivity. HNNs allow for better storage of multimodal data and can hold complex data connections.
Imbalanced regression: A regression problem where the target values are unevenly distributed, with some value ranges appearing much more frequently than others. As a result, models tend to perform well on common values but poorly on rare or extreme values, which may be the most important cases.
In-context learning (ICL): A capability of large language models where the model can perform a new task by using examples or instructions provided in the input context, without updating its parameters. The model learns patterns from the examples in the prompt and applies it to generate the desired output.^266^
Inverse-density kernels: Techniques to analyze, represent, or manipulate data distributions, often in reverse or in conjunction with density-based methods. These techniques are frequently used to handle nonparametric density estimations where traditional, rigid parametric models are unsuitable.
Junction tree: The transformation of any graph structure, directed or undirected, into an undirected graph where the nodes are summed into cliques. The formation of cliques allows for repetitive graph paths to be combined and the creation of a more concise graph.
Kernel methods: Machine-learning techniques that learn patterns by using a kernel function to measure similarity between data points, allowing models to operate in high-dimensional feature spaces without explicitly computing those features.
Latent space: A numerical representation space learned by a machine learning model, where each object is mapped to a *d*-dimensional vector that captures its key properties. Similar objects are placed closer together in this space, enabling tasks such as feature extraction, similarity search, and data generation.
Loss function: A function that tracks the degree of error in an AI model’s output by quantifying the difference (“loss”) between a predicted value (i.e., the model’s output) for a given input and the actual value (i.e., ground truth). A loss function indicates how well the model is performing.
Mask region-based CNN (Mask R-CNN): A subset of convolutional neural networks that allows for region-based comprehension of images, allowing for spatial understanding of image details as well as the possibility for bounding box generation. Mask R-CNN advances this framework by creating a pixel level representation of bounding box contents, allowing for segmentation of these pixels (mask) to occur.
Masked-token prediction (MTP): A self-supervised approach to infer missing tokens in a sequence or structured input given the surrounding context.
Message passing NN (MPNN): A type of neural network that utilizes node-to-node message passing to update node embeddings based on their neighbors. It is a type of graph-based ML architecture specifically designed to work directly on molecular structures, treating molecules as graphs where atoms are nodes and bonds are edges. MPNNs learn to “read” the molecular graph directly.
Meta-learning: The process of “learning to learn” within the AI space. Meta-learning trains models on a variety of tasks to acquire generalizable knowledge. This allows the model to leverage prior task experience and rapidly adapt to new tasks with minimal data requirements.
Monte Carlo tree search: A heuristic algorithm used for sequential decision making. Monte Carlo tree search relies on intelligent tree search that randomly samples a decision space (i.e., in the form of simulations), establishing node connections within that space (i.e., stores the statistics of actions, creating weights for nodes, regions of nodes, and node pathways) to make more educated choices in each subsequent iteration.
Neural classifier: A type of neural network utilized to identify input parameters as components of a predefined class (supervised classification) or as components of an unknown class (unsupervised classification).
Neural network (NN): A ML model, inspired by the human brain, consisting of multiple layers of interconnected computational units that contain information (neurons). Each layer processes the input and passes transformed information to the next layer, allowing the model to learn complex patterns, enabling tasks such as prediction, classification, and generation.
Nonautoregressive models: A class of ML models that can generate all outputs simultaneously, where each prediction is made independently of any sequential order. In molecular design or reaction prediction, these models can predict the entire molecular structure or reaction outcome as a SMILES string in one step, rather than generating it token by token.
Pretraining: The initial training stage in ML, where a model learns general patterns and representations from a large data set (often unlabeled). The pretrained model can then be fine-tuned on smaller, task-specific data sets for particular applications.
Prompting: The user-designed natural language input to a LLM that elicits a specific response or response format. Prompt-engineering, or the designing of prompts, allows for different utilizations of LLMs such as asking the model to perform a specific task, evaluate itself, or giving the model additional context/information before providing a query.
Proxy rewards: An imperfect reward function that approximately specifies the task to be learned. Proxy rewards are used in place of ground-truth rewards that are often difficult or impossible to define.
Reaction-center attention mechanisms: A type of model that concentrates on key functional groups, thereby generating potent representations for chemical reactions.
Recurrent NN (RNN): A type of neural network designed to process sequential data by carrying information from previous steps in the sequence. This allows the model to capture dependencies and patterns across the sequence, e.g., learning the syntax and structural patterns in SMILES strings for molecular property prediction.
Regression: A technique to forecast a continuous output based on a set of input features from data.
Reinforcement learning (RL): A learning framework where an agent (e.g., a neural network) learns to make decisions by interacting with an environment and receiving feedback in the form of rewards or penalties. The agent gradually improves its strategy (policy) to maximize the cumulative reward over time. When applied to molecule design, the model sequentially modifies molecular structures and receives rewards based on desired properties, such as drug-likeness, stability, or binding affinity, guiding it toward molecules with improved characteristics.
Sequence-to-sequence transformer (seq2seq): A ML model that maps one sequence to another, often used in prediction tasks. It uses an encoder-decoder architecture to connect two recurrent neural networks. These models often contain an attention mechanism that aligns output tokens with their correlated input token. In chemistry, these models can be used to convert molecular images into SMILES strings.^342^
Self-attention architecture: A neural network design where each element in a sequence can attend to and interact with all other elements when computing its representation. It is called “self” attention because the attention is computed within the same sequence. This allows the model to capture long-range dependencies and contextual relationships, as is the core mechanism used in Transformer models.
Self-supervised learning (SSL): A ML paradigm where a model learns useful representations from unlabeled data by creating its own training labels from the data itself. The model is trained to solve pretext tasks, such as predicting missing parts of the input or learning relationships within the data, which helps it capture underlying patterns that can later be used for downstream tasks.
Support vector machines (SVM): Statistical-based analytical models that study input data to develop pattern recognition techniques. This pattern recognition can then be used to assign labels to new data points.
Synthetic augmentation: A technique that creates new data points that mimic real data patterns, often without relying on existing samples.
Tanimoto similarity: A method for conducting molecular similarity using bit vectors where a fraction of features in common between two molecules are divided by the total features present in either molecule.
Token: A basic computational unit used by a model to represent text, such as a word, subword, character, or symbol. This unit, a unique digit, is converted into an embedding vector for model usage. In chemistry, tokens may correspond to atoms, bonds, or symbols in representations like SMILES strings.
Tokenization: The process of converting raw input data into a sequence of tokens that a model can process. Tokenization is particularly important in chemistry, as it determines how molecular representations (e.g., SMILES strings) are broken into meaningful units such as atoms, bonds, rings, or functional groups, which can strongly affect model performance.
Transformer architecture: A subclass of deep-learning models built around the self-attention mechanism to capture the relationship between tokens in a sequence. Transformers are composed of either an encoder, a decoder, or both, which allows for token production from individual or sequential input tokens.
Variational autoencoder (VAE): A type of ML model for constructing new data in the form of variations of the original input data they are trained on by a continuous, probabilistic representation of that latent space.
Vector hashing: Method for representing discrete data in a continuous vector space by projecting a value from a set with many components to a value from a set with a fixed number of (fewer) components.
Vision Transformer: A computer vision version of a transformer model. Vision transformers can be used for a variety of image processing tasks such as prediction, classification, object detection, and segmentation.
Zero-shot learning (ZSL): When a model is trained to recognize and categorize objects or concepts without having seen any examples of those categories or concepts beforehand during training.^507^
